# Transcriptional Control of Parallel-Acting Pathways That Remove Specific Presynaptic Proteins in Remodeling Neurons

**DOI:** 10.1523/JNEUROSCI.0893-20.2021

**Published:** 2021-07-07

**Authors:** Tyne W. Miller-Fleming, Andrea Cuentas-Condori, Laura Manning, Sierra Palumbos, Janet E. Richmond, David M. Miller

**Affiliations:** ^1^Neuroscience Program, Vanderbilt University, Nashville, Tennessee 37212; ^2^Department of Cell and Developmental Biology, Vanderbilt University, Nashville, Tennessee 37212; ^3^Department of Biological Sciences, University of Illinois at Chicago, Chicago, Illinois 60607

**Keywords:** *C. elegans*, development, presynaptic disassembly, synaptic plasticity, synaptic remodeling, transcriptional control

## Abstract

Synapses are actively dismantled to mediate circuit refinement, but the developmental pathways that regulate synaptic disassembly are largely unknown. We have previously shown that the epithelial sodium channel ENaC/UNC-8 triggers an activity-dependent mechanism that drives the removal of presynaptic proteins liprin-α/SYD-2, Synaptobrevin/SNB-1, RAB-3, and Endophilin/UNC-57 in remodeling GABAergic neurons in *Caenorhabditis elegans* (Miller-Fleming et al., 2016). Here, we report that the conserved transcription factor Iroquois/IRX-1 regulates UNC-8 expression as well as an additional pathway, independent of UNC-8, that functions in parallel to dismantle functional presynaptic terminals. We show that the additional IRX-1-regulated pathway is selectively required for the removal of the presynaptic proteins, Munc13/UNC-13 and ELKS, which normally mediate synaptic vesicle (SV) fusion and neurotransmitter release. Our findings are notable because they highlight the key role of transcriptional regulation in synapse elimination during development and reveal parallel-acting pathways that coordinate synaptic disassembly by removing specific active zone proteins.

**SIGNIFICANCE STATEMENT** Synaptic pruning is a conserved feature of developing neural circuits but the mechanisms that dismantle the presynaptic apparatus are largely unknown. We have determined that synaptic disassembly is orchestrated by parallel-acting mechanisms that target distinct components of the active zone. Thus, our finding suggests that synaptic disassembly is not accomplished by en masse destruction but depends on mechanisms that dismantle the structure in an organized process.

## Introduction

The nervous system is actively remodeled during development as new synapses are constructed and others are removed to refine functional circuits. Although synaptic assembly has been extensively investigated, synapse elimination is a less understood phenomenon despite its widespread occurrence (Goda and Davis, 2003; Südhof, 2018). In some cases, synaptic remodeling is limited to a specific developmental stage in which activity drives circuit plasticity. These “critical periods” are indicative of the necessary role of genetic programs that define specific developmental windows for activity-induced remodeling. Thus, synaptic remodeling mechanisms are likely to depend on the combined effects of both transcriptionally-regulated and activity-dependent pathways (Hensch, 2004; Hong and Chen, 2011; Kano and Watanabe, 2019).

**Figure 1.**
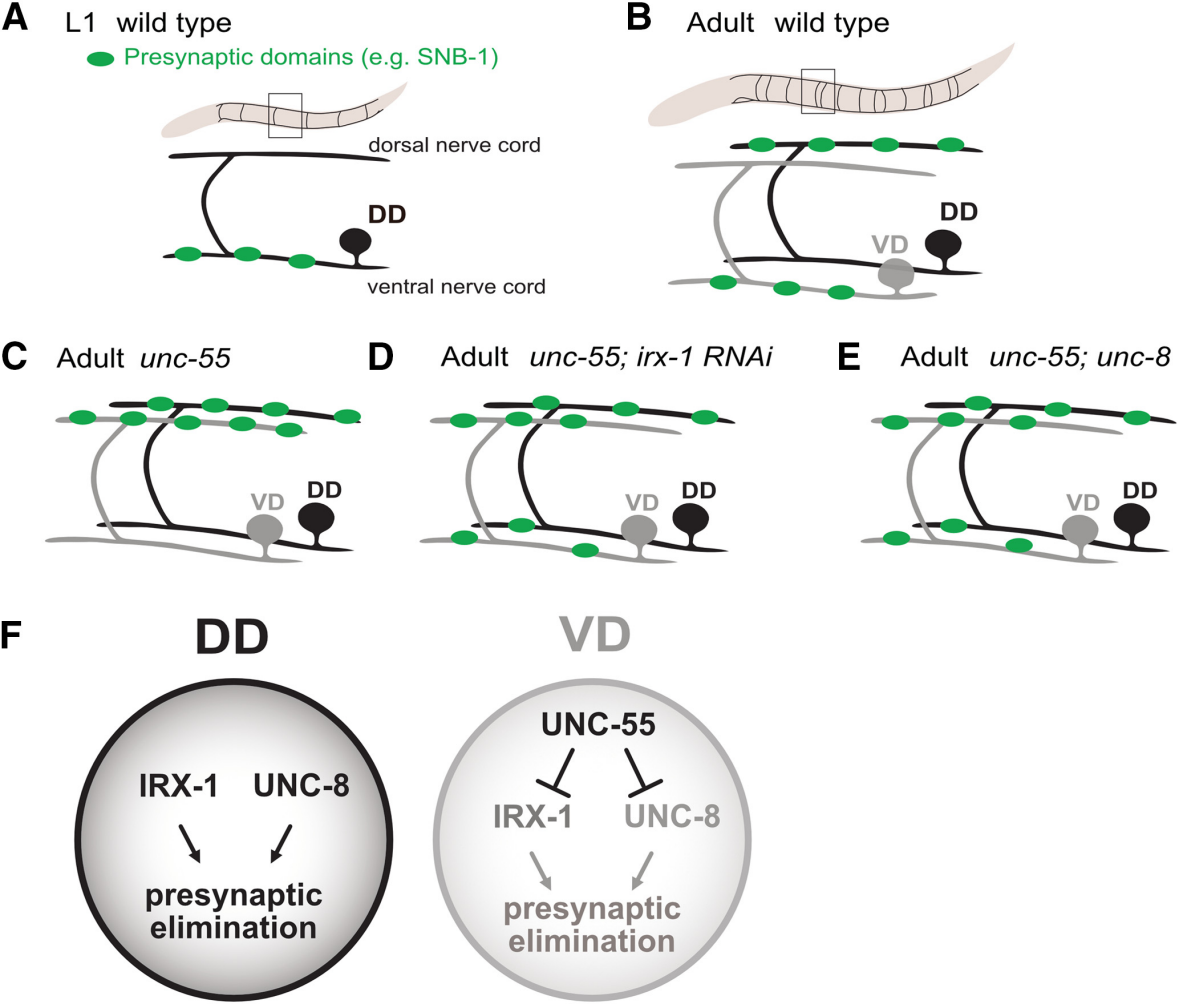
A transcriptional program regulates GABAergic neuron synaptic remodeling. ***A***, DD motor neurons innervate ventral muscles in early L1 stage larvae. GFP-tagged synaptobrevin (SNB-1::GFP; green puncta) marks GABAergic presynaptic domains. ***B***, DD synapses are relocated to the dorsal nerve cord during early larval development as postembryonic VD class GABAergic motor neurons are generated to synapse with ventral muscles. These DD and VD connections are maintained in the adult motor circuit. ***C***, In *unc-55* mutants, both DD and VD presynaptic domains are relocated to the dorsal nerve cord. ***D***, RNAi knock-down of the Iroquois family homeodomain transcription factor, IRX-1, antagonizes GABAergic neuron synaptic remodeling in *unc-55* mutants (Petersen et al., 2011). ***E***, Mutations that disable the DEG/ENaC cation channel, UNC-8, impair the removal of DD and VD GABAergic presynaptic domains in *unc-55* mutants (Miller-Fleming et al., 2016). ***F***, IRX-1 and UNC-8 are normally expressed in DD neurons to drive disassembly of the presynaptic apparatus. The COUP-TF transcription factor, UNC-55, blocks expression of IRX-1 and UNC-8 in VD neurons to prevent synapse elimination.

In *Caenorhabditis elegans*, synapses in the GABAergic motor circuit are relocated by a stereotypical remodeling program during early larval development (Cuentas-Condori and Miller, 2020). Dorsal D (DD) motor neurons are generated in the embryo and initially synapse with ventral body muscles (Fig. 1*A*). During the first larval stage, presynaptic domains are removed from ventral DD processes and then reassembled in the dorsal nerve cord (Fig. 1*B*; White et al., 1978; Hallam and Jin, 1998). Postembryonic ventral D (VD) neurons are born during this early larval period (Sulston, 1976) and synapse exclusively with ventral muscles (Fig. 1*B*). In the resultant mature circuit, GABAergic output alternates between dorsal (DD) and ventral (VD) muscles for sinusoidal movement (White et al., 1976, 1986).

The COUP-TF transcription factor, UNC-55, is selectively expressed in VD neurons to prevent synaptic remodeling (Zhou and Walthall, 1998; Shan et al., 2005), in *unc-55* mutants, VD neurons initially synapse with ventral muscles but then mimic the native DD remodeling program by relocating presynaptic domains to the dorsal nerve cord (Fig. 1*C*; Petersen et al., 2011; Thompson-Peer et al., 2012). The idea that UNC-55 normally blocks expression of genes that drive synaptic remodeling is supported by the finding that forced expression of UNC-55 in DD neurons is sufficient to prevent the native remodeling program (Shan et al., 2005). In earlier work, we exploited the synaptic remodeling phenotype of *unc-55* mutants in cell-specific profiling experiments to identify UNC-55 targets. An RNAi screen detected a subset of *unc-55*-regulated genes that are required for synaptic remodeling. For example, the homeodomain transcription factor, Iroquois/IRX-1, is ectopically expressed in *unc-55* mutant VD neurons which consequently undergo aberrant synaptic remodeling. RNAi knock-down of Iroquois/IRX-1, however, prevented the removal of GABAergic presynaptic domains from the ventral nerve cord in *unc-55* mutants (Fig. 1*D*; Petersen et al., 2011). Similarly, the DEG/ENaC cation channel subunit gene, *unc-8*, is upregulated in *unc-55* mutants and an *unc-8* loss-of-function allele also antagonized VD synaptic remodeling (Fig. 1*E*). Thus, these results argue that IRX-1 and UNC-8 are required for presynaptic elimination of remodeling GABAergic neurons. Additional experiments confirmed that both the Iroquois/IRX-1 and DEG/ENaC/UNC-8 normally promote the native DD remodeling program (Fig. 1*F*; Petersen et al., 2011; Miller-Fleming et al., 2016).

DEG/ENaC proteins function as cation channels and we have previously shown that UNC-8 gates sodium influx (Wang et al., 2013; Matthewman et al., 2016). The resultant membrane depolarization arising from UNC-8 channel activity is predicted to open local voltage-gated Ca^2+^ channels (VGCC) which we have shown function with UNC-8 to promote presynaptic disassembly. Based on these findings we have proposed that UNC-8 promotes presynaptic disassembly in a pathway that depends on intracellular calcium and neural activity (Miller-Fleming et al., 2016). Here, we show that DEG/ENaC/UNC-8 is transcriptionally-regulated by Iroquois/IRX-1 to remove a core group of presynaptic components including liprin-α/SYD-2, Synaptobrevin/SNB-1, RAB-3, and Endophilin/UNC-57. Surprisingly, proteins involved in synaptic vesicle (SV) priming, UNC-13/Munc-13 and ELKS, are not disassembled by UNC-8 but are removed by a separate pathway regulated by IRX-1/Iroquois. Together, these findings show that remodeling of GABAergic synapses depends on the combined effects of neural activity (UNC-8) and developmentally-regulated transcription (IRX-1). Thus, our work shows that synaptic disassembly can be orchestrated by parallel-acting mechanisms that selectively target molecularly distinct components of the presynaptic apparatus for removal.

## Materials and Methods

### Strains and genetics

*C. elegans* strains were cultured at either 20°C or 23°C as previously described on standard nematode growth medium seeded with OP50 (Brenner, 1974). The mutant alleles and strains used in this study are outlined in Tables 1, 2.

**Table 1.** Mutant alleles and genotyping primers used in this study

Allele	Source	Genotyping primer sequences
unc-8(tm5052) IV	NBRP	TGGGGCCCTAATAATTTCGA
		AGTGACAGTATGAAGCCAGG
unc-55(e1170) I	CGC	TAAGGACTACACGGATCCTG
		CCCAAGAAAGAAAAGAGAGGT
eri-1(mg366) IV	CGC	CATGCAATTTCAATGCCTTTTA
		TGCATCATCCAATCCACTATGT
unc-13(e51) I	CGC	TAGGCCTCCAAACGGACATA
		TGTCCTTCTTCGTAGCCTTC
unc-8(syb3726) IV	Sunybiotech	CTCCTACACCCTCTTCTGCT
		CCGAGCAAATGTTTCAACCT

**Table 2.** Strains used in this study

Strain	Genotype
Figure 2	
NC2758	*wdEx959 [pttr-39::irx-1 cDNA; pttr-39::irx-1 reverse; pttr-39::mcherry; str-1::GFP]*
NC2325	*unc-119; wdEx753[pttr-39::IRX-1::GFP; unc-119*+*]*
Figures 3, 4	
KP5348	*nuIs279 [punc-25::UNC-57::GFP;punc-25::mCherry::RAB-3]*
NC2984	*unc-55(e1170)* I; *nuIs279*
NC3279	*unc-55 (e1170)* I; *nuIs279*; *eri-1(mg366)* IV
NC2873	*unc-55(e1170)* I; *unc-8(tm5052)* IV; *nuIs279*
ZM54	*hpIs3[punc-25::SYD-2::GFP; lin-15*+*]* X
NC1849	*unc-55(e1170)* I; *hpIs3* X
NC1910	*unc-55 (e1170)* I; *eri-1 (mg366)* IV; *hpIs3* X
NC2874	*unc-55(e1170)* I; *unc-8(tm5052)* IV; *hpIs3* X
NC2388	*unc-55(e1170)* I*; unc-8(tm5052) juIs1* IV
Figure 5	
EG5052	*oxIs351[punc-47:ChR2::mCherry; lin-15*+ *LITMUS 38i]* X
NC2211	*unc-55(e1170)* I; *oxIs351* X
NC2807	*unc-55(e1170)* I; *unc-8(tm5052)* IV; *oxIs351* X
NC2212	*unc-55 (e1170)* I; *oxIs351* X; *wdEx686[irx-1 csRNAi]*
ZM1344	*hpIs61 [punc-25::UNC-10::GFP]* II
NC2872	*unc-55(e1170)* I; *hpIs61* II
NC2991	*unc-55(e1170)* I; *hpIs61* II; unc-8 (tm5052) IV
NC3337	*unc-55(e1170)* I; *hpIs61* II; *wdEx959* [*pttr-39*::irx-1 cDNA; *pttr-39*::irx-1 reverse; *pttr-39*::mcherry; *pstr-1*::GFP]
Figure 6	
NC2324	*unc-8 (tm5052)* IV
CB1170	*unc-55 (e1170*) I
NC3326	*unc-55 (e1170)* I; *unc-8 (tm5052)* IV
NC3342	*unc-55(e1170*) I*; unc-8(tm5052)* IV*; wdEx959*
Figure 7	
NC3330	*wdIs97 [punc-25::UNC-13L::GFP; pmyo-2::mCherry] ; unc-13(e51)* I
NC3216	*unc-55(e1170) unc-13(e51)* I; *wdIs97*
NC3341	*unc-55(e1170) unc-13(e51)* I; *unc-8 (tm5052)* IV; *wdIs97*
NC3339	*unc-55(e1170) unc-13(e51)* I; *wdIs97; wdEx959 [pttr-39::irx-1 cDNA; pttr-39::irx-1 reverse; pttr-39::mcherry; str-1::GFP]*
NC3511	*juIs1 [punc-25::SNB-1::GFP] IV; wdEx1104 [pttr39::UNC-8* cDNA*; punc-25::mCherry::RAB-3; pstr-1::GFP]*
NC3510	*unc-13(e51) I; wdIs105 [punc-25::UNC13L::GFP]; wdEx1102 [pttr39::UNC8cDNA; punc-25::mCherry::RAB-3; pstr-1::GFP]*
Figure 8	
KP5085	*nuIs249 [punc-25-ELKS-1::tdTomato; myo-3::NLS::GFP]*
NC3334	*unc-55(e1170)* I*; nuIs249*
NC3333	*unc-55(e1170)* I*; unc-8(tm5052)* IV*; nuIs249*
NC3708	*nuIs249; wdEx1160 [pttr-39::UNC-8*cDNA*; punc-25::GFP; pmyo-2::RFP]*
CZ13799	*juIs76 [unc-25p::GFP + lin-15(*+*)] II*
Figure 9	
NC3629	*rab-3 (ox785[GFP-FLPON::RAB-3 + loxp UNC-119*+ *loxp]) II; unc-19 III; wdEx1127 [pflp-13::flippase; pmyo-2::RFP]*
NC3683	*rab-3 (ox785) II; unc-8 (tm5052) IV; wdEx1127 [pflp-13::flippase; pmyo-2::RFP]*
NC3703	*rab-3 (ox785) II; wdEx1127 [pflp-13::flippase; pmyo-2::RFP]; wdEx959 [pttr-39::irx-1 cDNA; pttr-39::irx-1 reverse; pttr-39::mcherry; pstr-1::GFP]]*
NC3704	*rab-3 (ox785) II; unc-8(tm5052) IV; wdEx1127 [pflp-13::flippase; pmyo-2::RFP]; wdEx959 [pttr-39::irx-1 cDNA; pttr-39::irx-1 reverse; pttr-39::mcherry; pstr-1::GFP]]*
Figure 10	
NC3696	*elks-1(wy1162, frt::PEST-degron::operon::frt = GFP::flp-on at N-terminus) IV; wdEx1127 [pflp-13::flipasse; pmyo-2::RFP]*
NC3702	*elks-1(wy1162) IV; wdEx1127 [pflp-13::flipasse;pmyo-2::RFP]; wdEx959[pttr- 39::irx-1 cDNA; pttr-39::irx-1 reverse; pttr-39::mcherry; pstr-1::GFP]*
C3803	*elks-1(wy1162) unc-8(syb3726) IV; wdEx1127 [pflp-13::flipasse; pmyo-2::RFP]*

### Microscopy

#### Confocal microscopy

Larval or young adult animals were immobilized on 2% agarose pads with 15 mm levamisole as previously described (Smith et al., 2010). Z-stack images (Figs. 3*A*,*D*, 7*A–F*, 8*A–F*) were collected on a Leica TCS SP5 confocal microscope using a 63× oil objective (0.5 μm/step), spanning the focal depth of the ventral nerve cord GABA neurons and synapses. Leica application suite advanced fluorescence (LAS-AF) software was used to generate maximum intensity projections. Ventral nerve cord images were straightened using an ImageJ plug-in. Z-stack images (Figs. 4, 5*I*, 9, 10) were acquired with Nikon confocal A1R using Apo Fluor 40×/1.3 and 60×/1.4 N.A. oil objective (0.3 μm/step).

#### Image analysis

Synapse density counts (Figs. 3*A–F*, 7*G*, 8*G*) were collected by tracing segments of the ventral nerve cord using the segmented line tool in ImageJ (VD3–VD11). Distance in micrometers and gray value plot traces were used to count the number of peaks (synapses) that occur over the specified distance. Synapses were defined as fluorescent peaks that reached a threshold of 25 arbitrary units of fluorescence intensity. See example in Figure 3*B*,*C*.

NIS Elements 5.2 software was used to produce Figures 4, 8, 9. Synaptic density for each marker (SYD-2::GFP, RAB-3::mCherry, UNC-57::GFP, UNC-10::GFP, endogenous GFP::RAB-3, and endogenous GFP::ELKS-1) was calculated using the General Analysis tool. First, images were preprocessed to subtract background using Rolling Ball Correction. Then, the intensity threshold was defined for each marker and binary objects were filtered by size and circularity. Each object along the nerve cord was considered a synaptic punctum. Density was defined as the number of puncta per 10 μm of dendrite.

FIJI was used to quantify effects of UNC-8(OE) (overexpression) on presynaptic disassembly (Figs. 7, 8). Z-stacks were collected for the full length of the ventral nerve cord. In Figure 7*H–K*, mCherry-positive VD cells carry an UNC-8 cDNA transgenic array (Miller-Fleming et al., 2016). Neighboring mCherry-positive [e.g., UNC-8(OE)] and mCherry-negative (control) VD neurons were compared with quantify differences in the fluorescence signal for SNB-1::GFP and UNC-13L::GFP arisng from UNC-8 overexpression. In Figure 8*J*,*K*, GFP-positive VD neurons carry an UNC-8 cDNA transgenic array. Neighboring GFP-positive [e.g., UNC-8(OE)] and GFP-negative (control) VD neurons were compared with quantify differences in the fluorescence signal. Because unc-8 cDNA transgenic arrays are mosaic with expression limited to a random subset of VD neurons in each animal, data were collected from VD neurons (VD3–VD11) carrying the UNC-8 cDNA (mcherry-positive for Fig. 7*H–K* or GFP-positive for Fig. 8*J*,*K*) versus an adjacent control VD neuron that does not carry the array. For results shown in Figures 7*H–K*, 8*J*,*K*, intensity values were obtained from line scans anterior to the VD cell bodies of interest. Background fluorescence was obtained from a line scan of an adjacent region inside the animal and subtracted from the VD line scans.

### Single-molecule mRNA fluorescence *in situ* hybridization (smFISH)

**Figure 2.**
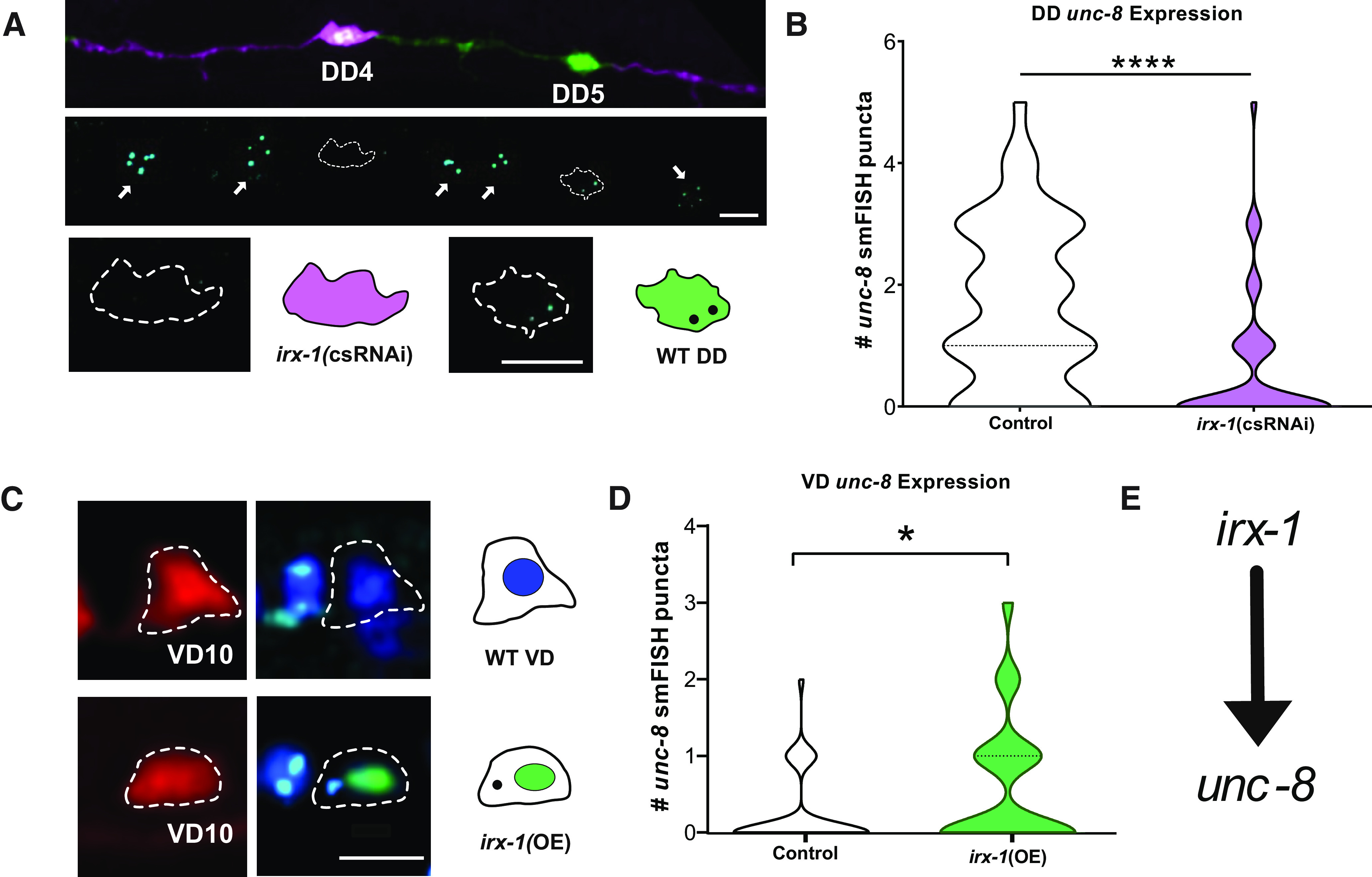
IRX-1 drives expression of UNC-8/DEG/ENaC in GABAergic neurons. ***A***, *irx-1(csRNAi)* blocks *unc-8* expression in DD neurons. Top, Mosaic expression of *irx-1(csRNAi)* (magenta) in DD4 versus adjacent control DD5 neuron (green) in the L1 ventral nerve cord. Middle, smFISH puncta for *unc-8* transcripts (cyan). Dashed lines demarcate DD cell soma and arrows denote *unc-8* smFISH puncta in adjacent cholinergic motor neurons. Bottom, Dashed outlines and graphical representations depict *irx-1(csRNAi)-*marked DD4 neuron (magenta) and *unc-8* smFISH puncta in control DD5 neuron (green). Scale bar: 5 µm. ***B***, Quantification of *unc-8* smFISH puncta in DD neurons. Violin plots for *unc-8* smFISH puncta in control (white; *n* = 49) versus *irx-1(csRNAi)* (magenta; *n* = 50) in L1 stage DD motor neurons. Dashed line represents median. Mann–Whitney test, *p* < 0.0001. ***C***, In the wild type, VD neurons do not express *irx-1* or *unc-8* but forced expression of IRX-1 in VD neurons [*irx-1(OE)*] is sufficient to activate *unc-8* transcription. Representative images of VD neurons (VD10) in control (top) versus *irx-1(OE)* VD neurons (bottom). Left, Dashed lines denote VD cell soma marked with mCherry. Right, DAPI (dark blue) labels the nucleus. *unc-8* smFISH probe (cyan puncta). Note IRX-1::GFP (green) labels VD10 nucleus in *irx-1(OE)* (bottom right). Scale bar: 5 µm. ***D***, Quantification of *unc-8* smFISH puncta in VD motor neurons. Violin plots for *unc-8* smFISH puncta in control (white; *n* = 40) and *irx-1(OE)* (green; *n* = 50) in L3 stage VD neurons. Dashed line represents median. Mann–Whitney test, *p* = 0.0184. ***E***, Working model: IRX-1 promotes UNC-8 expression in GABAergic motor neurons.

*smFISH* was performed with custom *unc-8* probes linked to Quasar 670 (Biosearch Technologies). Synchronized larvae (from either late L1 or early L3 stage) were collected by washing plates with M9, fixed in 4% paraformaldehyde in 1× PBS for 45 min and permeabilized in 70% ethanol for 48 h. Hybridization followed the manufacturer's instructions (http://www.biosearchtech.com/stellarisprotocols) and was performed at 37°C for 16 h in Stellaris RNA FISH hybridization buffer (Biosearch Technologies catalog #SMF-HB1-10) containing *unc-8* probe at 1:100. For *irx-1* cell-specific RNAi (csRNAi; Fig. 2*A*), all DD motor neurons were marked with *Punc-47*::GFP (*oxIs12*) and specific DDs expressing the *irx-1(csRNAi)* constructs (*pttr-39::irx-1* sense, *pttr-39::irx-1* antisense) were co-labeled with *Punc-25::*mCherry to distinguish them from DD neurons that did not express the *irx-1(csRNAi)* transgenic array. For *IRX-1(OE)* (IRX-1 overexpression) experiments (Fig. 2*C*), GFP-tagged IRX-1 was expressed with the *ttr-39* promotor (*pttr-39::IRX-1::*GFP). In this setup, VDs and DDs were marked with *Punc-47::mCherry* (*wpIs39*) and individual DDs or VDs expressing IRX-1(OE) were detected by nuclear-localized IRX-1::GFP (Petersen et al., 2011). For UNC-8(OE) (overexpression) experiments (Fig. 8*H*,*I*), UNC-8 was expressed in DD and VD neurons (*pttr39::*UNC-8; Miller-Fleming et al., 2016) from a transgenic array also expressing p*unc-25*::GFP to label DD and VD neurons and compared with wild-type DD and VD neurons in an *punc-25::*GFP marker strain (*juIs76*; Huang et al., 2002). In all cases, cell nuclei were stained with DAPI. Z-stacks were collected in a Nikon spinning disk confocal microscope with optical filters for DAPI, Quasar 670, and GFP using an Apo TIRF 100× objective (NA = 1.49) in 0.2-μm steps spanning the cell body and merged for quantification following 3D-deconvolution in NIS elements. smFISH puncta were defined in Nikon Elements as circular fluorescent spots (circularity filter) that exceeded the Quasar 670 background signal (e.g., fluorescence threshold). To confirm localization within DD/VD cell soma, only puncta that co-localized with either GFP (Figs. 2*A*, 8*H*) or mCherry (Fig. 2*C*) labeled DD/VD cell bodies in both X-Y and Z axes were counted. At least 30 worms were scored for each group and the Mann–Whitney test used to determine significance (*n* > 45 neurons). As a positive control, *unc-8* smFISH staining was noted in adjacent DA and DB ventral-cord neurons for all samples to confirm successful hybridization.

### Electron microscopy (EM)

Young adult hermaphrodites of each strain were prepared for high-pressure freeze (HPF) fixation as described (Rostaing et al., 2004; Miller-Fleming et al., 2016). A total of 10–15 animals were loaded into a specimen chamber filled with *Escherichia coli*. The specimens were frozen rapidly in a high-pressure freezer (Leica HPM100) at −180°C and high pressure. Freeze substitution was performed on frozen samples in a Reichert AFS machine (Leica) with 0.1% tannic acid and 2% OsO_4_ in anhydrous acetone. The temperature was kept at −90°C for 107 h, increased at 5°C/h to −20°C, and kept at −20°C for 14 h. The temperature was then increased by 10°C/h to 20°C. Fixed specimens were embedded in Epon resin after infiltration in 50% Epon/acetone for 4 h, 90% Epon/acetone for 18 h, and 100% Epon for 5 h. Embedded samples were incubated for 48 h at 65°C. All specimens were prepared using the same fixation procedure and labeled with anonymous tags so that the examiner was blinded to genotype. Ultrathin (40 nm) serial sections were cut using an Ultracut 6 (Leica) and collected on formvar-covered, carbon-coated copper grids (EMS, FCF2010-Cu). Grids were counterstained in 2% aqueous uranyl acetate for 4 min, followed by Reynolds lead citrate for 2 min. Images were obtained on a Jeol JEM-1220 transmission EM operating at 80 kV. Micrographs were collected using a Gatan digital camera at a magnification of 100,000. Images were quantified using NIH ImageJ software. Dorsal and ventral cords were distinguished by size and morphology. GABAergic synapses were identified by previously established criteria, including position in the cord as well as the morphology of the synapse. GABAergic synapses are larger than their cholinergic motor neuron counterparts, and the active zones in these synapses form a direct, perpendicular angle with muscle arms. In contrast, the presynaptic density in cholinergic synapses orient at an acute angle to the muscle, generally 30–45° and are often dyadic. Some images were collected at 30k to aid in identifying synaptic identity based on terminal position in the cord. Two colleagues with expertise in EM reconstruction of the *C. elegans* ventral nerve cord independently reviewed synapse images from each strain to verify identification. Each profile represents an image of a 40-nm section. A synapse was defined as a set of serial sections containing a presynaptic density with two flanking sections either side without presynaptic densities. SVs were identified as spherical, light gray structures with an average diameter of ∼30 nm. SVs were considered docked if they were in direct contact with the membrane. Three to five animals were imaged for each genotype. Numbers of profiles for each genotype were (# analyzed/# imaged): wild type = 80/1330, *unc-55; unc-8* = 37/745, *unc-55;irx-1(csRNAi)* = 54/613 for ventral GABAergic synapse evaluation.

### Electrophysiology

The *C. elegans* dissection and electrophysiological methods were as previously described (Richmond and Jorgensen, 1999; Miller-Fleming et al., 2016). Animals were immobilized along the dorsal axis with Histoacryl Blue glue, and a lateral cuticle incision was made with a hand-held glass needle, exposing ventral medial body wall muscles. Muscle recordings were obtained in the whole-cell voltage-clamp mode using an EPC-10 patch-clamp amplifier and digitized at 1 kHz. The extracellular solution consisted of 150 mm NaCl, 5 mm KCl, 5 mm CaCl_2_, 4 mm MgCl_2_, 10 mm glucose, 5 mm sucrose, and 15 mm HEPES (pH 7.3, ∼340 mOsm). The intracellular solution consisted of 120 mm KCl, 4 mm KOH, 4 mm MgCl_2_, 5 mm *N*-tris[hydroxymethyl] methyl-2-aminoethane-sulfonic acid, 0.25 mm CaCl_2_, 4 mm Na_2_ATP, 36 mm sucrose, and 5 mm EGTA (pH 7.2, ∼315 mOsm). GABAergic miniIPSCs and hyperosmotic responses were acquired at a holding potential of −60 mV by pressure-ejecting extracellular saline containing an additional 500 mOsm of sucrose; 10 mm d-tubocurare (dTBC) was added to both the extracellular solution and the pressure ejection pipette to block cholinergic hyperosmotic currents. Data were acquired using Pulse software (HEKA) on a Dell computer. Subsequent analysis and graphing were performed using Pulsefit (HEKA), Mini analysis (Synaptosoft Inc.) and Igor Pro (Wavemetrics).

### Molecular biology

#### Generation of the punc-25::UNC-13L::GFP transgenic line

We used the In-Fusion cloning kit (Takara) to amplify the cDNA of the long isoform of UNC-13 (UNC-13L) from a plasmid provided by J. Kaplan (pTWM88). This fragment was ligated into a vector containing the *punc-25* GABA promoter and a C-terminal GFP tag. The resulting plasmid, pTWM90, was injected into *unc-13 (e51)* null mutants at 25 ng/µl with the co-injection marker *pmyo-2::mCherry* (2 ng/µl). This transgenic array was integrated by x-ray irradiation and outcrossed for three generations to generate stable transgenic lines for analysis.

#### Generation of the pflp-13::flippase transgenic line

We used the In-Fusion cloning kit (Takara) to amplify the flippase sequence from a plasmid provided by the Jorgensen lab pMLS262 (Addgene #73718). This fragment was ligated into a vector containing the *pflp-13* DD promoter. The resulting plasmid, pACC98, was injected at 25 ng/µl with the co-injection marker *pmyo-2::mCherry* (2 ng/µl) generating the array *wdEx1127*. This array was later crossed with *rab-3(ox785)*, *elks-1(wy1162)*, and *elks-1(wy1162) unc-8(syb3726)* to activate GFP-tagging in DD neurons.

#### Generation of unc-8 (syb3726) in wy1162 animals

Sunybiotech used CRISPR/Cas9 to generate a deletion allele, *unc-8(syb3726)*, in *wy1162* [*elks-1* FLP-ON GFP] animals. *unc-8(syb3726)* removes 6977 nucleotides from the R13A1.4b.1 *unc-8* transcript except for the first 8 base-pairs and the last 44 nucleotides and thus is a likely null allele.

### Feeding RNA interference experiments

Bacteria producing either double-stranded *irx-1* RNA or containing the RNAi empty vector were seeded on NGM plates and stored at 4°C for up to one week. Four L4 *unc-55*, *unc-55; eri-1*, or *unc-55; unc-8* animals were grown on each single RNAi plate at 23°C until progeny reached the L4 stage. Progeny were picked to fresh RNAi plates and the ventral synapses were quantified.

### Movement assays

Animals were first tapped on the tail to ensure that they were capable of forward locomotion, then tapped on the head to assess ability to execute backward locomotion. Animals were binned into the following categories: unc (uncoordinated: coil ventrally immediately on tapping), initiate backing (initiate backwards movement but stop), and wild type (sustain backward locomotion with at least two body bends). In Figure 6, the wild type and initiate backing categories were grouped into a single initiate backing category.

### IRX-1 csRNAi

The *irx-1(csRNAi)* array (*wdEx959*) was outcrossed from NC2975 (He et al., 2015) and combined with synaptic markers displayed in Figures 5, 7, 9, 10 using conventional genetic methods.

### Experimental design and statistical analysis

For samples that are not normally distributed, the Mann–Whitney test was used to compare two groups and determine significance. For comparisons between three or more groups, we used the Kruskal–Wallis test with multiple comparison. For samples that are normally distributed, Student's *t* test was used to compare two groups and one-way ANOVA with Bonferroni correction for multiple comparisons among more than two groups. Figure legends specify the statistical test used in each case and the number of independent measurements (*N*) evaluated.

## Results

### The homeodomain transcription factor, iroquois/IRX-1, drives DEG/ENaC/UNC-8 expression in remodeling GABAergic neurons

In previous work, we used gene expression profiling and an RNAi screen to identify protein-encoding genes that promote presynaptic disassembly in remodeling GABAergic neurons (Petersen et al., 2011). These studies determined that the homeobox transcription factor Iroquois/IRX-1 and the DEG/ENaC ion channel subunit UNC-8 promote removal of the presynaptic vesicular SNARE protein, Synaptobrevin/SNB-1 (Fig. 1*D*,*E*; Petersen et al., 2011; Miller-Fleming et al., 2016). Because both IRX-1/Iroquois and DEG/ENaC/UNC-8 are expressed in remodeling DD neurons, we investigated the hypothesis that Iroquois/IRX-1 functions as a transcription factor to regulate DEG/ENaC/UNC-8 expression. First, we used smFISH to confirm expression of *unc-8* transcripts in remodeling (control) DD neurons (Fig. 2). We note that *unc-8* is highly expressed in adjacent DA and DB cholinergic neurons as previously reported (Wang et al., 2013; Miller-Fleming et al., 2016). We then targeted *irx-1* in DD neurons with csRNAi (see Materials and Methods) and detected significantly fewer *unc-8* transcripts in comparison to untreated (control) DD neurons (Fig. 2*A*,*B*). These results are consistent with the idea that Iroquois/IRX-1 is required for DEG/ENaC/UNC-8 expression in DD neurons.

GFP reporters for *irx-1* and *unc-8* are not expressed in wild-type VD neurons which normally do not remodel. However, forced expression of Iroquois/IRX-1 in VD motor neurons is sufficient to trigger VD remodeling and drive the elimination of VD presynaptic terminals (Petersen et al., 2011; Miller-Fleming et al., 2016). Thus, we next asked whether Iroquois/IRX-1 overexpression [*irx-1(OE)*] could also induce *unc-8* expression in VD neurons. smFISH quantification confirmed that *unc-8* transcripts are elevated in *irx-1(OE)* VD neurons in comparison to controls (Fig. 2*C*,*D*). Together, these results demonstrate that the transcription factor, Iroquois/IRX-1, is both necessary and sufficient for DEG/ENaC/UNC-8 expression in remodeling GABA neurons (Fig. 2*E*).

### Iroquois/IRX-1 drives a DEG/ENaC/UNC-8-dependent mechanism of presynaptic disassembly

In addition to promoting the removal of Synaptobrevin/SNB-1::GFP from ventral presynaptic domains in remodeling GABAergic neurons (Fig. 1), UNC-8 also drives the elimination of RAB-3/GTPase, liprin-α/SYD-2 and endophilin/UNC-57 (Petersen et al., 2011; Miller-Fleming et al., 2016). RAB-3/GTPase interacts with SVs for exocytosis (Fischer von Mollard et al., 1990; Nonet et al., 1997), liprin-α/SYD-2 is a scaffolding protein that defines the presynaptic dense projection area (Zhen and Jin, 1999; Stigloher et al., 2011; Kittelmann et al., 2013), and endophilin/UNC-57 mediates SV endocytosis and recycling (Schuske et al., 2003; Yu et al., 2018).

**Figure 3.**
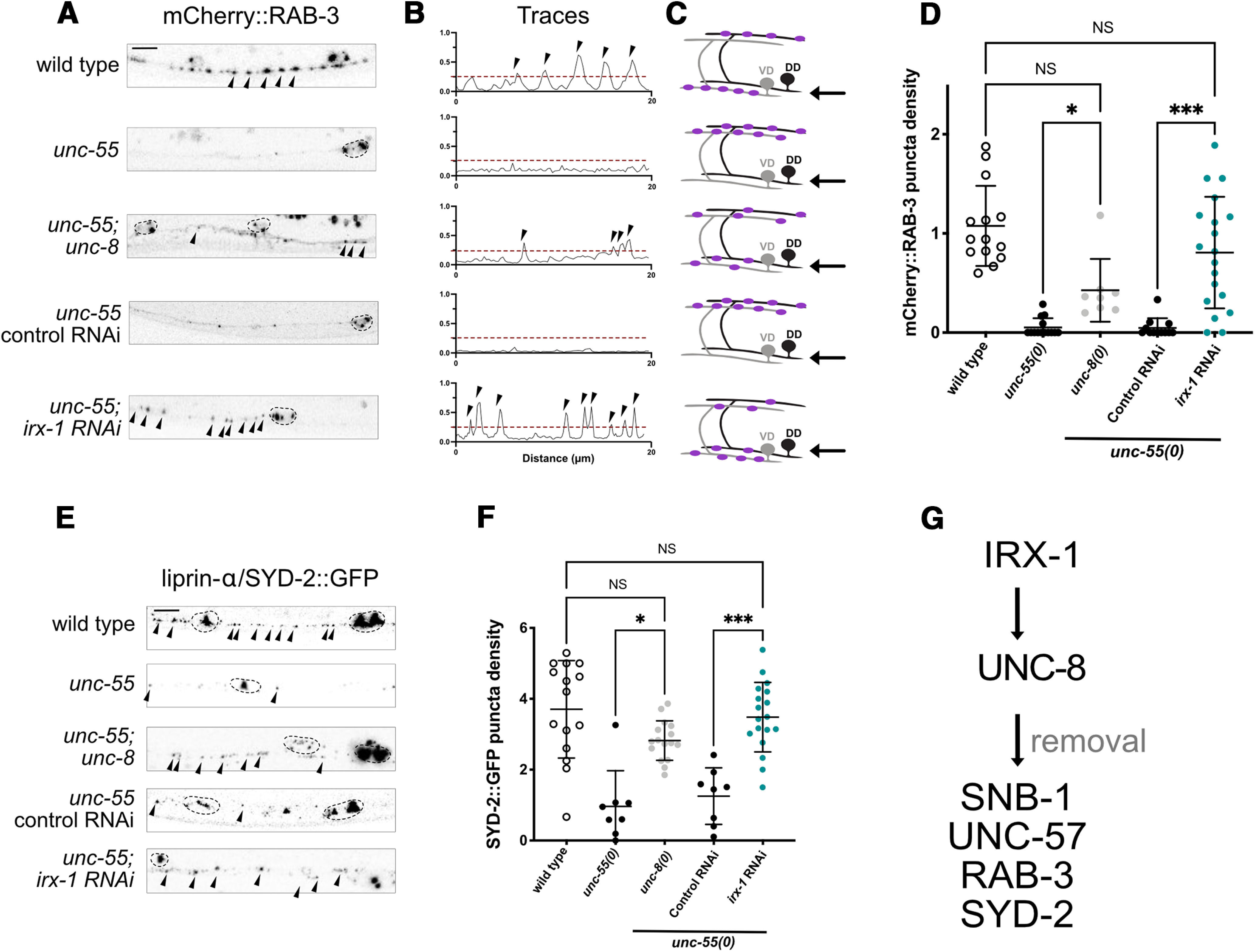
IRX-1/Iroquois drives UNC-8 expression to dismantle the GABAergic presynaptic apparatus. ***A***, Representative images of RAB-3::mCherry-labeled GABA neuron synapses (arrowheads) in the ventral nerve cord of adult animals. Note that RAB-3::mCherry puncta are removed from the ventral nerve cord of *unc-55* mutants in which both DD and VD neurons remodel (see Fig. 1*C*) but are partially retained in *unc-55; unc-8.* Similarly, residual RAB-3::mCherry puncta are detectable in *unc-55; irx-1* RNAi but not in unc-55 RNAi control animals. ***B***, Representative calculation of peak number (puncta). Line scans were drawn along the ventral nerve cord of each animal on the left. Peaks that passed a 25% threshold (dashed line) were counted (arrowheads). ***C***, Schematics of mCherry::RAB-3 puncta in each genotype. Arrow denotes ventral region depicted in panels ***A***, ***B***. ***D***, RAB-3::mCherry puncta density quantified for each genotype: wild type (1.07 ± 0.4), *unc-55; unc-8* (0.43 ± 0.3 puncta/10 μm), and *unc-55; irx-1(RNAi)* (0.81 ± 0.6 puncta/10 μm) show more ventral RAB-3::mCherry-marked puncta than *unc-55* (0.05 ± 0.1 puncta/10 μm) and *unc-55* control RNAi (0.05 ± 0.1 puncta/10 μm). Data are mean ± SD; *p < 0.05, ***p < 0.001. NS is not significant. Kruskal–Wallis test with multiple comparison because *unc-55*, *unc-55; unc-8*, and *unc-55* control RNAi samples are not normally distributed; *N* > 8 animals. ***E***, Representative images of liprin-α/SYD-2::GFP-labeled GABA neuron synapses (arrowheads) in the ventral nerve cord. Dashed lines demarcate DD and VD cell soma. ***F***, SYD-2::GFP puncta density (puncta/10 μm) quantified for each genotype: wild type (3.7 ± 1.4), *unc-55* (0.97 ± 1.0), *unc-55; unc-8* (2.82 ± 0.6), *unc-55* control RNAi (1.26 ± 0.8) and *unc-55; irx-1(RNAi)* (3.48 ± 0.9). Data are mean ± SD; *p < 0.05, ***p < 0.001. NS is not significant. Kruskal–Wallis test with multiple comparison because *unc-55* data are not normally distributed; *N* > 8 animals. All images of L4 stage larva, anterior to left. Arrowheads denote GABA neuron presynaptic puncta. Scale bars:10 μm. VD3–VD11 were scored on the ventral side for all genotypes. ***G***, Working model: the presynaptic components, Synaptobrevin/SNB-1, Endophilin/UNC-57, Rab3/RAB-3, and Liprin-α/SYD-2 are dismantled by IRX-1 and UNC-8.

If Iroquois/IRX-1 activates UNC-8 expression as predicted by our smFISH results (Fig. 2), then Iroquois/IRX-1 should also promote removal of these additional presynaptic markers. To test this possibility, we exploited *unc-55* mutants in which the VD GABAergic presynaptic domains are eliminated because of ectopic activation of the native DD remodeling program (Zhou and Walthall, 1998; Fig. 1*C*) (Tables 1, 2). In this paradigm, removal of ventral GABAergic synapses in *unc-55* mutants is prevented by mutations that disable the pro-remodeling program (Petersen et al., 2011). For example, ventral mCherry::RAB-3 puncta are largely eliminated from GABAergic synapses in *unc-55* mutant animals but a significant fraction is retained in *unc-55; unc-8* double mutants (Fig. 3*A*). This result indicates that UNC-8 function is required for the efficient removal of presynaptic RAB-3 in remodeling GABAergic neurons (Miller-Fleming et al., 2016). As an upstream activator of *unc-8* expression, Iroquois/IRX-1 is also predicted to remove mCherry::RAB-3 from ventral synapses of *unc-55* mutants. As expected, RNAi knock-down of *irx-1* prevents the elimination of ventral RAB-3::mCherry puncta in *unc-55* mutants (Fig. 3*A–D*). Additional experiments showed that ablation of either *unc-8* or *irx-1* also blocks the removal of SYD-2::GFP (Fig. 3*E*,*F*) in the ventral nerve cord of *unc-55* mutants. Since Iroquois/IRX-1 induces UNC-8 expression (Fig. 2), these results are consistent with the hypothesis that Iroquois/IRX-1 drives an UNC-8-dependent mechanism to remove presynaptic terminals in GABAergic neurons (Fig. 3*G*).

### Iroquois/IRX-1 drives a separate parallel-acting remodeling pathway that does not require UNC-8 for synaptic removal

**Figure 4.**
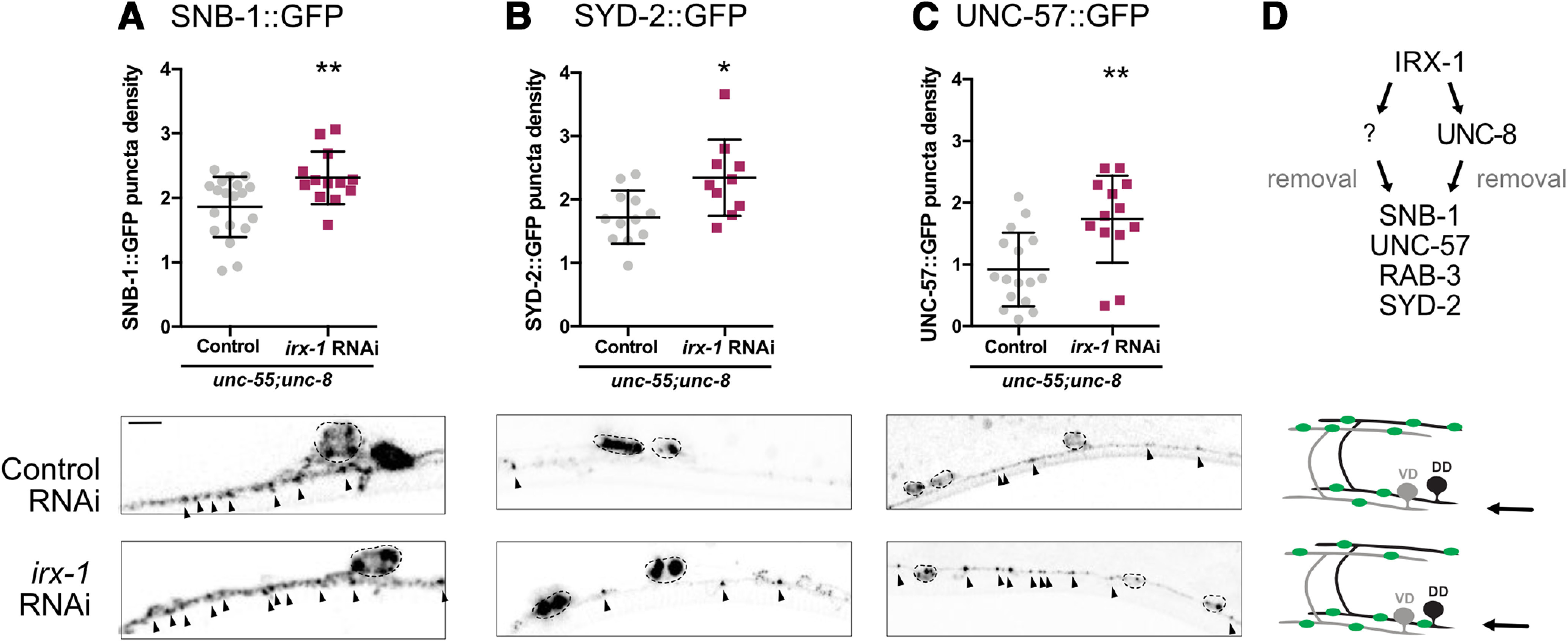
IRX-1/Iroquois activates parallel-acting pathways that remove presynaptic components. ***A***, bottom, Representative images of *irx-1*-RNAi-treated *unc-55; unc-8* mutants show more ventral SNB-1::GFP (2.31 ± 0.4 puncta/10 μm) than *unc-55; unc-8* controls (1.86 ± 0.5 puncta/10 μm). Data are mean ± SD; *N* > 12. Unpaired *t* test; **p = 0.0086. ***B***, *irx-1* RNAi-treated *unc-55; unc-8* mutants show more SYD-2::GFP (2.34 ± 0.6 puncta/10 μm) than *unc-55; unc-8* controls (1.72 ± 0.4, puncta/10 μm). Data are mean ± SD; *N* > 9. Unpaired *t* test; *p = 0.01. ***C***, *irx-1* RNAi-treated *unc-55; unc-8* mutants show more UNC-57::GFP (1.73 ± 0.7 puncta/10 μm) than *unc-55; unc-8* controls (0.92 ± 0.6 puncta/10 μm). Data are mean ± SD; *N* > 12. Unpaired *t* test; **p = 0.0023. VD3–VD11 were scored on the ventral side for all genotypes. ***D***, Working model: IRX-1 activates UNC-8 expression and an additional parallel-acting pathway (?) to remove Synaptobrevin/SNB-1, Endophilin/UNC-57, Rab3/RAB-3 and Liprin-α/SYD-2 from remodeling GABAergic presynaptic terminals.

Because *irx-1* encodes a transcription factor, we reasoned that Iroquois/IRX-1 might also regulate other targets in addition to the *unc-8* gene in the GABA neuron synaptic remodeling pathway. If Iroquois/IRX-1 regulates a downstream target that functions in tandem with UNC-8, then genetic ablation of *irx-1* should enhance the retention of ventral presynaptic markers in *unc-55; unc-8* double mutants. For this test, we used feeding RNAi for global knock-down of *irx-1* because the *irx-1* null allele is lethal (Petersen et al., 2011). The *unc-8(tm5052)* deletion allele used for these experiments is a likely null mutation (Miller-Fleming et al., 2016). We counted GFP puncta for the presynaptic proteins SNB-1::GFP, SYD-2::GFP and UNC-57::GFP in *unc-55; unc-8* double mutants versus *unc-55; unc-8* animals treated with *irx-1-*RNAi. This experiment revealed that RNAi knock-down of *irx-1* increases the number of ventral SNB-1::GFP, SYD-2::GFP, and UNC-57::GFP puncta (Fig. 4*A–C*) in *unc-55; unc-8* double mutant animals. Together, these results suggest that Iroquois/IRX-1 drives an additional genetic pathway, independent of UNC-8, that also eliminates presynaptic terminals in remodeling GABAergic neurons (Fig. 4*D*). In the next series of experiments, we used a combination of ultrastructural analysis, electrophysiology and genetics to confirm that Iroquois/IRX-1 functions in tandem with *unc-8* to dismantle the presynaptic apparatus.

### Iroquois/IRX-1 and DEG/ENaC/UNC-8 dismantle the presynaptic apparatus in remodeling GABAergic neurons

**Figure 5.**
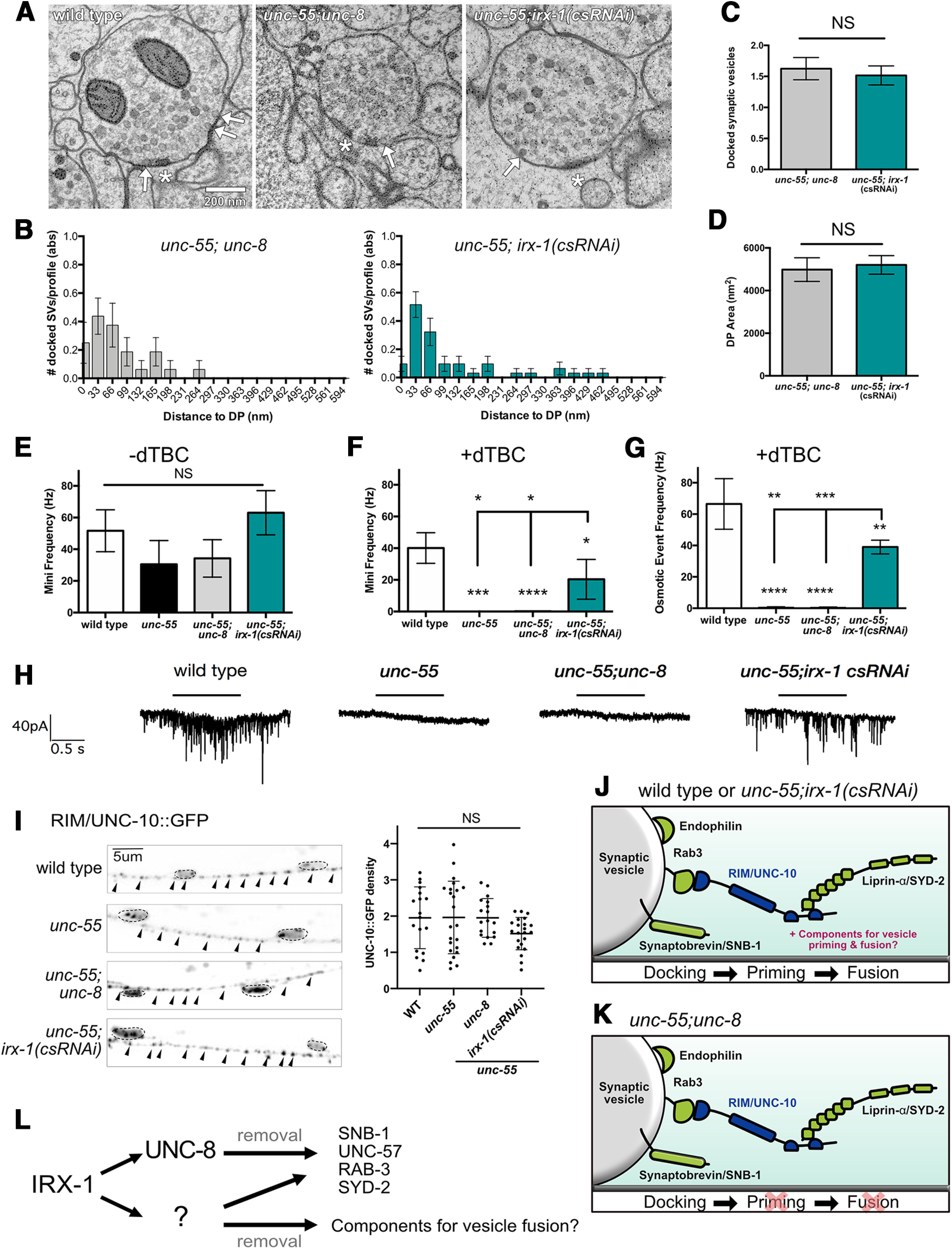
The transcription factor IRX-1/Iroquois removes presynaptic components required for SV fusion and GABA release. ***A***, Representative electron micrographs of GABAergic motor neuron synaptic terminals with ventral muscles for wild type, *unc-55; unc-8*, and *unc-55*; *irx-1(csRNAi)*. Asterisks denote presynaptic density. Arrows point to docked SVs. Scale bar: 200 nm. ***B***, Distribution of docked SVs per profile plotted in bins denoting distance from the dense projection (DP) of *unc-55; unc-8* (left) and *unc-55; irx-1(csRNAi)* animals (right). A Kruskal–Wallis test detected no significant difference between *unc-55; unc-8* versus *unc-55; irx-1(csRNAi)* in the distribution of docked vesicles either <100 nm (*p* = 0.52) or >100 nm (*p* = 0.13) from the DP. ***C***, Numbers of docked SVs are similar between *unc-55; unc-8* (1.62 ± 0.18), and *unc-55; irx-1(csRNAi)* (1.52 ± 0.15) animals. Data are Mean ± SEM. Non-parametric Mann–Whitney test, *p* = 0.2664. A total of 16 synaptic profiles were evaluated for *unc-55; unc-8* double mutants and 31 synaptic profiles for *unc-55; irx-1(csRNAi).* NS = not significant. ***D***, DP area is not significantly different between *unc-55; unc-8* (4981 ± 555.7 nm^2^, *n* = 31 profiles) and *unc-55; irx-1(csRNAi)* (5201 ± 435.4 nm^2^, *n* = 32 profiles) animals. Data are mean ± SEM. Unpaired *t* test, *p* = 0.378. NS = not significant. ***E***, ***F***, Endogenous miniPSCs (both GABA and ACh minis are inward under these recording conditions) obtained from body wall muscles voltage-clamped at –60 mV, before (***E***) and after (***F***) dTBC application, remaining minis in ***F*** represent GABA minis. ***G***, GABA release in response to hyperosmotic saline in the presence of dTBC were eliminated in *unc-55* (0 ± 0) and *unc-55; unc-8* animals (0 ± 0.25). In contrast, *unc-55; irx-1(csRNAi)* (39.0 ± 2.5) partially restored hyperosmotic release, wild type (66.5 ± 8.1); *N* ≥ 3, data are mean ± SEM. One-way ANOVA Bonferroni correction, ***p = 0.001, **p = 0.01. ***H***, Representative traces showing that ventral mini-iPSCs are detected for wild-type and *unc-55; irx-1(csRNAi)* animals, but not for *unc-55* or *unc-55; unc-8* mutants. Horizontal lines denote hyperosmotic treatment which fails to evoke mini-iPSCs in either *unc-55* or *unc-55; unc-8* mutants. ***I***, Left, Representative images of UNC-10::GFP-labeled GABA neuron synapses (arrowheads) in the ventral cord of wild-type, *unc-55, unc-55;unc-8*, and *unc-55;irx-1(csRNAi)* animals. Dashed lines demarcate DD and VD cell soma. Scale bar: 10 µm. Right, Ventral density (puncta/10 μm) of UNC-10::GFP is not different between wild-type (WT; 1.97 ± 0.9, *n* = 18), *unc-55* (2.05 ± 1.2, *n* = 23), *unc-55; unc-8* (1.95 ± 0.5, *n* = 18), and *unc-55; irx-1* (csRNAi; 1.51 ± 0.4, *n* = 22) animals. Data are mean ± SD. One-way ANOVA, *p* = 0.149. NS is not significant across individual pairs between all genotypes. ***J***, In wild-type and *unc-55; irx-1(csRNAi)* animals, SVs can dock, prime, and fuse, which suggests that these synapses include additional components that are required for neurotransmitter release. ***K***, In *unc-55; unc-8* animals, SVs can dock but do not fuse with the presynaptic membrane to release GABA. ***L***, Working model: IRX-1 activates UNC-8 expression to remove structural components of the presynaptic apparatus. IRX-1 drives a parallel acting pathway (?) that also dismantles presynaptic proteins that are required for vesicle fusion and release.

We previously used EM to establish that GABAergic presynaptic terminals are removed in *unc-55* mutants as predicted from experiments showing that presynaptic markers (e.g., mCherry::RAB-3, SYD-2::GFP) are eliminated (Walthall and Plunkett, 1995; Fig. 3). EM analysis also confirmed that ventral GABAergic presynaptic domains are retained in *unc-55; unc-8* adults, as expected since ectopic UNC-8 drives the elimination of ventral presynaptic markers in remodeling GABAergic neurons (Miller-Fleming et al., 2016). Since our assays with fluorescent presynaptic markers also showed that Iroquois/IRX-1 drives presynaptic disassembly (Figs. 3, 4), we used EM to ask whether csRNAi knock-down of *irx-1* would prevent the removal of ventral GABAergic synapses in an *unc-55* mutant. These experiments detected GABAergic presynaptic terminals in *unc-55; irx-1(csRNAi)* samples (Fig. 5*A*), thus, confirming that IRX-1 is necessary for the removal of presynaptic domains in remodeling GABA neurons.

GABAergic synapses in *unc-55; unc-8* and *unc-55; irx-1(csRNAi)* animals are strikingly similar to wild-type GABAergic presynaptic domains (Fig. 5*A*) with normal numbers of docked SVs (Fig. 5*B*,*C*). Previous work has shown that SV docking at the presynaptic active zone depends on the vesicular GTPase protein RAB-3 and the RAB-3-Interacting Molecule, RIM1/UNC-10 (Weimer et al., 2006; Gracheva et al., 2008). RAB-3 is largely absent from the ventral processes of GABAergic neurons in *unc-55* animals as a result of ectopic VD remodeling (Thompson-Peer et al., 2012; Miller-Fleming et al., 2016), but is at least partially restored in *unc-55; unc-8* and *unc-55; irx-1(csRNAi)* animals (Fig. 3*A*; Miller-Fleming et al., 2016). We additionally determined that fluorescently-labeled RIM1/UNC-10 localizes to ventral GABAergic synapses in *unc-55; unc-8* and *unc-55; irx-1(csRNAi)* animals (Fig. 5*I*). Surprisingly, unlike other presynaptic markers, Rim1/UNC-10::GFP does not remodel in *unc-55* mutants and is retained on the ventral side (Fig. 5*I*). Together, these results suggest that dual localization of both RAB-3 and Rim1/UNC-10 in ventral GABAergic synapses of *unc-55; unc-8* and *unc-55; irx-1(csRNAi)* mutants could account for our EM observation of numerous docked vesicles (Fig. 5*B*,*C*). In addition, the ventral synapses detected in *unc-55; unc-8* and *unc-55; irx-1*(*csRNAi)* animals show comparable dense projections (Fig. 5*D*). This characteristic presynaptic structure has been previously shown to depend on SYD-2 (Zhen and Jin, 1999; Stigloher et al., 2011; Kittelmann et al., 2013) and its normal appearance in *unc-55; unc-8* and *unc-55; irx-1(csRNAi)* GABAergic synapses is consistent with the persistence of ventral SYD-2::GFP in these animals (Fig. 3*E*,*F*, Fig. 4*B*). Taken together, our EM data are consistent with the independent findings that SNB-1, UNC-57, RAB-3 and SYD-2 puncta (Figs. 3, 4; Petersen et al., 2011; Miller-Fleming et al., 2016) are retained in *unc-55; unc-8* and *unc-55; irx-1(csRNAi)* animals and thus support the hypothesis that functional UNC-8 and IRX-1 proteins are required for the removal of the ventral presynaptic apparatus in remodeling GABAergic neurons motor neurons.

### Iroquois/IRX-1 removes fusion-competent SVs in remodeling GABAergic neurons

Because ventral GABAergic synapses with apparently normal ultrastructure are observed in *unc-55; unc-8* and *unc-55; irx-1(csRNAi)* (Fig. 5*A–D*), we next performed experiments to determine whether these synapses are functional. We first examined whether the docked SVs in *unc-55; unc-8* animals are fusion-competent by recording iPSCs from ventral muscles. For these experiments, we used dTBC to block cholinergic signaling. As previously reported, tonic release of ventral iPSCs was restored in *unc-55; irx-1(csRNAi)* animals, but iPSCs were not detected in *unc-55; unc-8* mutants despite the presence of organized clusters of fluorescent presynaptic proteins (Fig. 3), electron dense active zones and docked vesicles in both strains (Fig. 5*A–D*; Petersen et al., 2011; Miller-Fleming et al., 2016). Previously, we determined that the postsynaptic GABA_A_ receptor UNC-49 was also properly localized and functional in *unc-55;unc-8* animals (Miller-Fleming et al., 2016), thus excluding the possibility that the absence of iPSCs in *unc-55; unc-8* animals is because of a postsynaptic defect. The lack of tonic release in *unc-55; unc-8* animals could be because of defective vesicle priming or downstream Ca*^2+^-*sensing. To determine whether the morphologically-docked SVs in *unc-55; unc-8* animals are primed, we measured iPSCs in response to hypertonic sucrose perfusion which is sufficient to induce neurotransmitter release from the primed SV pool (Fig. 5*E–H*). Hyperosmotic treatment of *unc-55; unc-8* animals failed to trigger ventral GABA release suggesting that these animals are defective in SV priming (Fig. 5*G*,*H*). As expected, hyperosmotic stimulation triggered robust iPSCs in wild-type and in *unc-55; irx-1(csRNAi)* animals, whereas ventral muscles in *unc-55* mutants were unresponsive. To rule out the possibility of a general defect in SV fusion, we confirmed that preparations of *unc-55* and *unc-55; unc-8* mutants exhibited spontaneous cholinergic activity that could be abolished by dTBC (Fig. 5*E*,*F*). Together with our EM results (Fig. 5*A–D*; Miller-Fleming et al., 2016), these data indicate that SVs at ventral GABAergic synapses in *unc-55; unc-8* animals are capable of docking with the presynaptic membrane but are not fusion competent. We further conclude that SVs at ventral GABAergic synapses in *unc-55; irx-1(csRNAi)* animals can dock and are also fusion competent since endogenous miniature IPSCs were detected and hyperosmotic treatment evoked robust IPSCs in *unc-55; irx-1(csRNAi)* adults (Fig. 5*F–H*). Thus, our results are consistent with the idea that Iroquois/IRX-1 and UNC-8 eliminate a shared set of presynaptic proteins (e.g., RAB-3, Synaptobrevin/SNB-1) in the remodeling program but that Iroquois/IRX-1 selectively removes specific presynaptic components for vesicle priming and neurotransmitter release in a mechanism that does not require UNC-8.

### A behavioral assay for functional GABAergic synapses in the motor circuit

**Figure 6.**
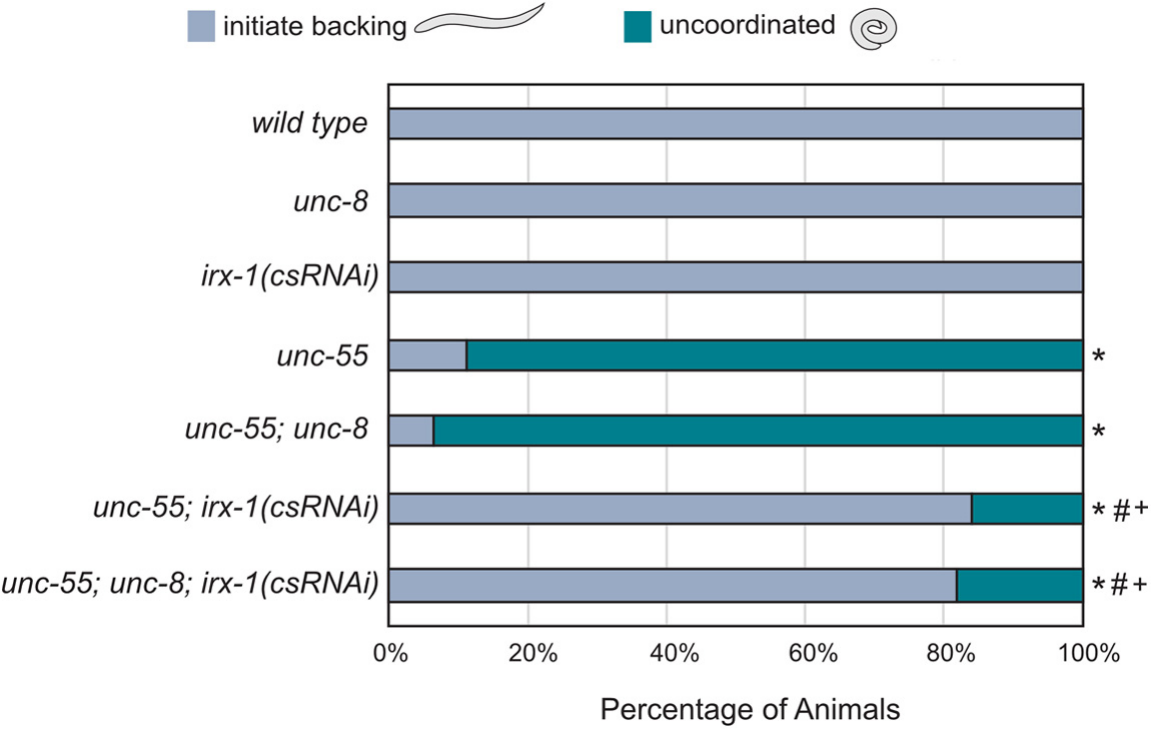
A behavioral assay for functional GABAergic synapses in the motor circuit. Behavioral assays to detect backward locomotion. Young adult animals were tapped on the head and scored for wild type (gray) versus uncoordinated (teal) backward movement. *unc-55* and *unc-55; unc-8* animals coil ventrally with head tap indicating that a loss-of-function *unc-8* mutation does not rescue backward locomotion in *unc-55* mutants (NS, not significant; Fisher's exact test, *n* ≥ 100 animals per genotype). *csRNAi* knock-down of *irx-1* restores backward locomotion to *unc-55* animals and this effect is not enhanced in *unc-55; unc-8; irx-1(csRNAi)*; * significantly different from wild type with *p* < 0.002; # significantly different from *unc-55* with *p* < 0.002; + significantly different from *unc-55; unc-8* with *p* < 0.002.

As an additional test of our ultrastructural and electrophysiological results, we devised a behavioral assay to evaluate the functionality of GABAergic synapses in remodeling defective mutants (Fig. 6). Ventral synapses for both DD and VD neurons are dismantled in *unc-55* mutants and reassembled in the dorsal nerve cord (Fig. 1*D*). The resultant imbalance of excess inhibitory GABAergic output to dorsal muscles versus excess excitatory cholinergic input to ventral muscles results in a striking behavioral phenotype in which *unc-55* animals coil ventrally when tapped on the head instead of initiating coordinated backward locomotion (Walthall and Plunkett, 1995; Shan et al., 2005). We have shown that *irx-1(csRNAi)* restores fusion-competent GABAergic presynaptic densities to *unc-55* mutants (Fig. 5*G*,*H*). If these restored synapses are functional, then the tapping assay should detect improved backward locomotion. Indeed, *unc-55; irx-1(csRNAi)* animals (Fig. 6) show robust backward movement in comparison to *unc-55* mutants (Petersen et al., 2011). This result is congruent with our previous finding that GABAergic release is restored to ventral cord synapses of *unc-55* mutants by RNAi knock-down of *irx-1* (Fig. 5*F*–*H*). In contrast, *unc-55; unc-8* mutant animals show severely defective backward locomotion that is not significantly different from that of *unc-55* mutants (Fig. 6). This finding is in agreement with our observation that hyperosmotic treatment fails to evoke GABA release (Fig. 5*G*,*H*) and reinforces the idea that ventral GABAergic synapses in *unc-55; unc-8* mutants are not functional. Genetic ablation of *unc-8* activity in *unc-55; irx-1(csRNAi)* does not further enhance backward locomotion (Fig. 6) as predicted by our conclusion that residual ventral cord GABAergic synapses in *unc-55; unc-8* double mutants are dysfunctional (Fig. 5*F*,*G*) and by our finding that Iroquois/IRX-1 regulates expression of the *unc-8* gene (Fig. 2). To summarize, the results of the behavioral assay suggest that although ultrastructurally normal ventral GABAergic synapses are visible by EM in both *unc-55; unc-8* and in *unc-55; irx-1(csRNAi)* animals (Fig. 5*A*), GABAergic release is selectively reactivated by knock-down of *irx-1*, but not by genetic removal of *unc-8* (Fig. 5*F*-*H*). This striking difference suggests that Iroquois/IRX-1 must drive the removal of key determinants of presynaptic neurotransmitter release that are not targeted by UNC-8.

**Figure 7.**
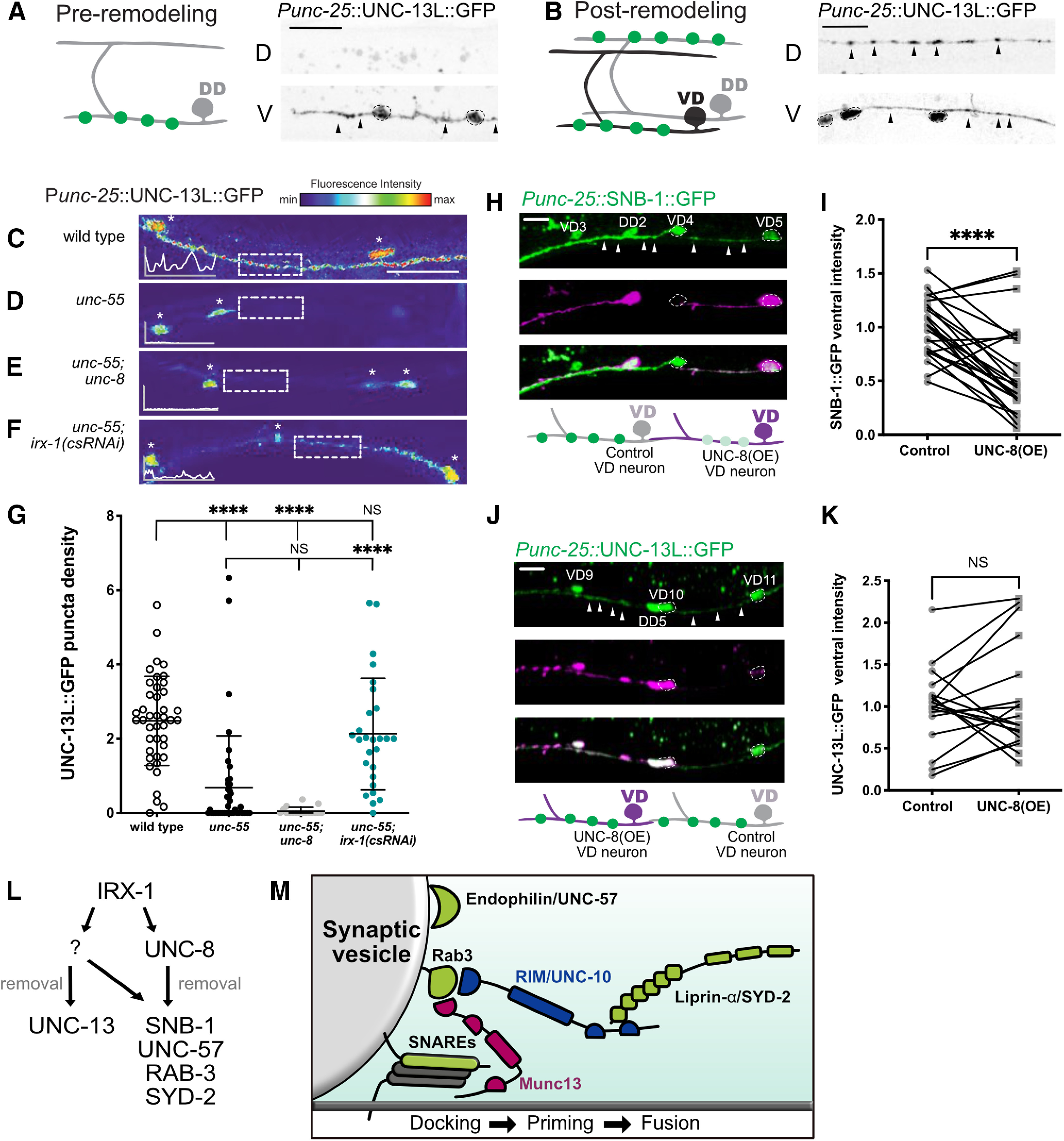
IRX-1/Iroquois, but not UNC-8, drives removal of Munc13/UNC-13 from the presynaptic domains of remodeling GABAergic neurons. ***A***, GFP-tagged UNC-13L (*Punc-25::*UNC-13L::GFP) is expressed in GABAergic neurons and localized to the ventral (V) nerve cord before DD remodeling in early L1 larvae. Scale bar: 5 µm. ***B***, UNC-13L::GFP is visible in both the dorsal (D) and ventral (V) nerve cords after DD remodeling at the L4 stage. Arrowheads mark UNC-13L::GFP puncta and dashed circles denote GABA neuron cell soma. Scale bar: 10 µm. ***C–F***, Representative fluorescence intensity heat maps and line tracings of UNC-13L::GFP-labeled GABA neuron synapses in the ventral nerve cord of (***C***) wild type, (***D***) *unc-55*, (***E***) *unc-55; unc-8*, and (***F***) *unc-55; irx-1(csRNAi)*. UNC-13L::GFP signal is depleted in (***D***) *unc-55* and (***E***) in *unc-55; unc-8*, but partially restored in (***F***) *unc-55; irx-1(csRNAi)* (boxes with dashed lines). Asterisks denote GABAergic neuron cell soma. Scale bar: 10 μm. ***G***, Quantification of UNC-13L::GFP (puncta/10 μm) at ventral GABAergic synapses (VD3–VD11) for wild type (2.48 ± 1.2), *unc-55* (0.68 ± 1.4), *unc-55; unc-8* (0.05 ± 0.1), and *unc-55; irx-1(csRNAi)* (2.19 ± 1.5). Data are mean ± SD; *N* > 16. Kruskal–Wallis test with multiple comparison because *unc-55* and *unc-55;unc8* samples are not normally distributed; ****p < 0.0001. NS = not significant. ***H***, top, Representative image of SNB-1::GFP in ventral nerve cord of mosaic animal with forced UNC-8 expression [UNC-8(OE)] in a subset of VD neurons (magenta, VD5, dashed outline) versus neighboring control cells (VD4, dashed outline). Arrowheads denote regions of SNB-1::GFP signal in control and UNC-8(OE) VD neurons. Below, Schematic depicting brighter SNB-1::GFP signal (green) in control (gray) versus UNC-8(OE) VD neurons (magenta). Scale bar: 10 μm. ***I***, Forced expression of UNC-8 in VD neurons (1.00 ± 0.3) reduces SNB-1::GFP in GABAergic terminals [UNC-8(OE)] compared with neighboring control VD neurons that do not express UNC-8 (0.58 ± 0.4); *N* = 24 animals. Paired *t* test; ****p < 0.0001. ***J***, top, Representative image of UNC-13L::GFP in ventral nerve cord of mosaic animals with forced UNC-8 expression [UNC-8(OE)] in a subset of VD neurons (magenta, VD10, dashed outline) versus neighboring control cells (VD11, dashed outline). Arrowheads point to examples of UNC-13L::GFP signal in control and UNC-8(OE) VD neurons. Below, Schematic of UNC-13L signal (green) in control (gray) and UNC-8(OE) cells (magenta). Scale bar: 10 μm. ***K***, Forced expression of UNC-8 in VD neurons (1.00 ± 0.5) does not reduce UNC-13L::GFP in ventral VD neuron GABAergic terminals [UNC-8(OE)] compared with neighboring control VD neurons that do not express UNC-8 (1.08 ± 0.6). Data are mean ± SD; *N* = 18. NS = not significant; *p* = 0.723. Paired *t* test. All images from L4 animals, anterior to left. ***L***, Working model: IRX-1 promotes an *unc-8*-independent pathway involving unknown downstream components (?) that removes UNC-13L from remodeling GABAergic synapses. ***M***, IRX-1 knock-down blocks remodeling of GABAergic synapses which retain presynaptic structural components (green), RIM/UNC-10 (blue), and Munc13 (magenta) allowing docked vesicles to prime and fuse with the plasma membrane. Also depicted are the plasma-membrane SNAREs (gray) because these are required for functional synapses, which are removed by IRX-1 (Fig. 5*G*).

### Iroquois/IRX-1, but not DEG/ENaC/UNC-8, removes the SV priming protein UNC-13 in remodeling GABAergic neurons

The cytosolic protein Munc13/UNC-13 functions as a conserved component of the presynaptic apparatus to mediate SV fusion (Brose et al., 1995; Augustin et al., 1999; Richmond et al., 1999; Kohn et al., 2000; Weimer et al., 2006; Südhof, 2012). Mammalian neurons express four UNC-13-related proteins whereas only two distinct UNC-13 proteins, a long (UNC-13L) and a short (UNC-13S) version, are expressed in *C. elegans*. Because SVs appear docked, but are incapable of fusion in *unc-55; unc-8* mutants (Fig. 5*A–D*, *F–H*), we hypothesized that UNC-13 could be absent from ventral GABAergic synapses in these animals. To test this idea, we generated a strain expressing GFP-tagged UNC-13L protein in GABA neurons. We selected UNC-13L for this experiment because it co-localizes with UNC-10/RIM whereas the short isoform, UNC-13S, shows a diffuse distribution in GABAergic motor neurons (data not shown), as also reported for *C. elegans* cholinergic motor neurons (Hu et al., 2013). As previously observed for other presynaptic proteins (Fig. 3; Hallam and Jin, 1998; Petersen et al., 2011; Thompson-Peer et al., 2012; Miller-Fleming et al., 2016), UNC-13L::GFP is restricted to the ventral nerve cord before DD remodeling in early L1 larvae, but is detectable postremodeling in both the dorsal and ventral nerve cords in adults (Fig. 7*A*,*B*). This finding indicates that UNC-13L::GFP remodels to DD presynaptic domains in the dorsal nerve cord and is also a component of ventral VD synapses in the adult. We quantified the number of UNC-13L::GFP puncta in the ventral nerve cord and determined that UNC-13L::GFP is largely removed in *unc-55* mutants (Fig. 7*D*,*G*) thus demonstrating that UNC-13L::GFP is disassembled from the ventral presynaptic domains of DD neuron and also VD neurons that undergo remodeling in *unc-55* mutants. In contrast to other presynaptic markers (e.g., SNB-1::GFP; Fig. 3*A–C*), UNC-13L::GFP is also eliminated from ventral GABAergic synapses in *unc-55; unc-8* mutants (Fig. 7*E*,*G*). Thus, wild-type UNC-8 activity is not required for the removal of UNC-13L from remodeling GABAergic synapses. This finding suggests that although the ventral presynaptic active zone in *unc-55; unc-8* mutant GABAergic neurons appears normal by EM (Fig. 5*A–D*; Miller-Fleming et al., 2016), UNC-13L is not localized at these terminals thus likely accounting for their SV fusion defect (Fig. 5*E–G*).

Since UNC-8 expression in VD neurons was shown to drive elimination of SNB-1::GFP (Miller-Fleming et al., 2016), we devised an additional experiment to test the idea that removal of UNC-13L is UNC-8 independent. We confirmed that forced expression of UNC-8 in VD neurons is sufficient to remove SNB-1::GFP from ventral GABAergic synapses (Fig. 7*H*,*I*), but does not displace UNC-13L::GFP (Fig. 7*J*,*K*). Together, these results show that UNC-8 function is neither necessary nor sufficient for UNC-13L removal from remodeling GABAergic synapses.

RNAi knock-down of *irx-1* in *unc-55* mutants is sufficient to restore ventral GABAergic synaptic release (Fig. 5*F–H*). Thus, we next asked whether UNC-13L::GFP is retained in the ventral nerve cord of *unc-55; irx-1(csRNAi)* animals. Indeed, we found that ventral UNC-13L::GFP puncta are detectable in both wild-type and in *unc-55; irx-1(csRNAi)* animals (Fig. 7*F*,*G*), thus indicating that Iroquois/IRX-1 is required for the removal of UNC-13L from remodeling GABAergic synapses (Fig. 7*L*,*M*).

### Iroquois/IRX-1, but not DEG/ENaC/UNC-8, removes the RIM-binding protein (RBP) ELKS-1 in remodeling GABAergic neurons

**Figure 8.**
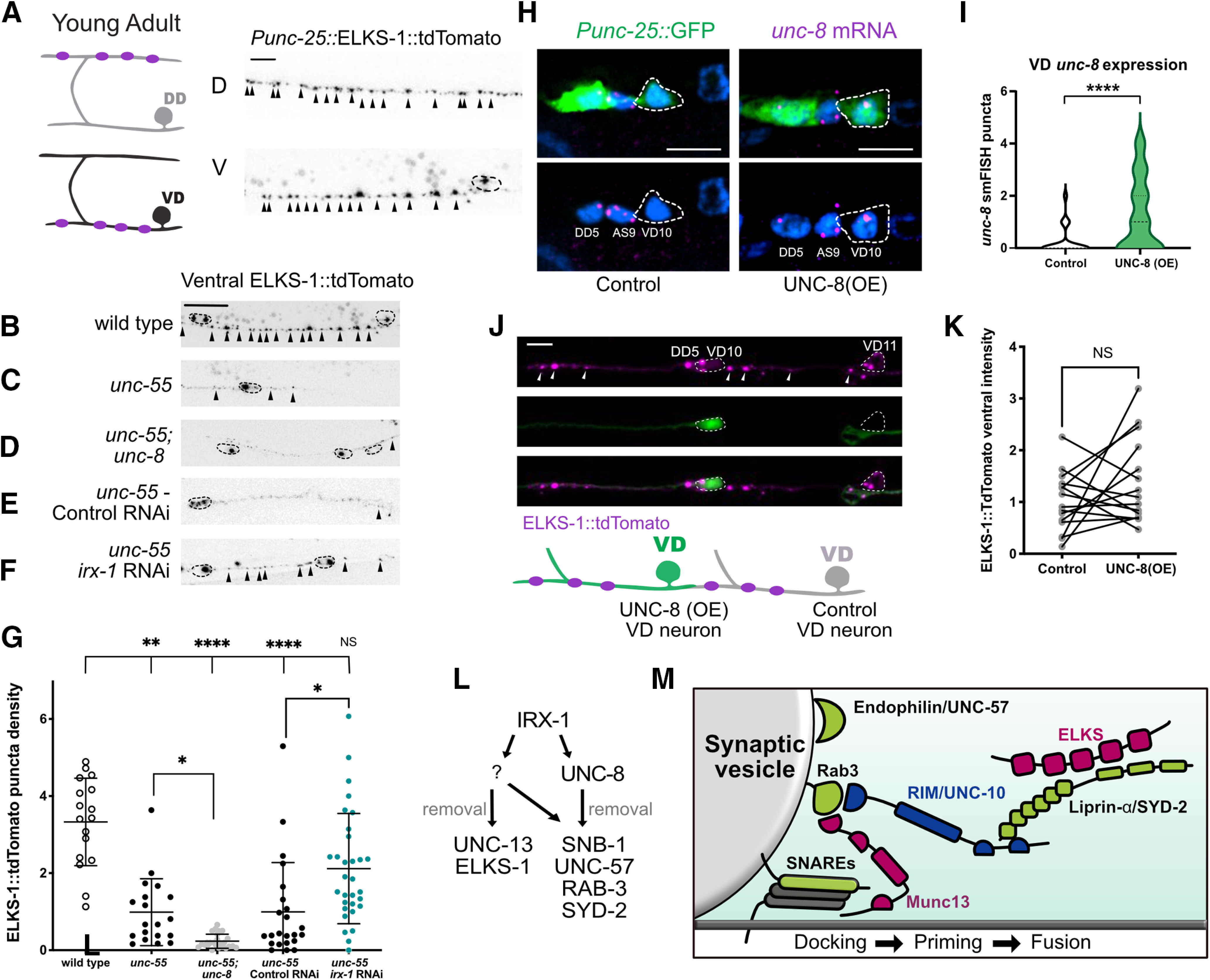
IRX-1, but not UNC-8, drives removal of ELKS-1 from the presynaptic domains of remodeling GABAergic neurons. ***A***, ELKS-1::TdTomato puncta (magenta) are visible in both the dorsal (D) and ventral (V) nerve cords after DD remodeling at the L4 stage. Arrowheads point to ELKS-1::TdTomato puncta and dashed circles demark cell soma. Scale bar: 5 µm. ***B–F***, Representative images of ELKS-1::TdTomato-labeled GABA neuron synapses in the ventral nerve cord of (***B***) wild type, (***C***) *unc-55*, (***D***) *unc-55; unc-8*, (***E***) *unc-55* control RNAi, (***F***) *unc-55; irx-1* RNAi. Arrowheads point to ELKS-1::TdTomato puncta and dashed circles demark cell soma. GABAergic ELKS-1::TdTomato ventral puncta are largely absent in (***C***) *unc-55*, (***D***) *unc-55; unc-8*, and (***E***) *unc-55*; control RNAi animals but are restored with (***F***) *irx-1* RNAi treatment of *unc-55* mutants. Scale bar: 10 μm. Images from L4 larvae, anterior to left. ***G***, Quantification of ventral GABAergic synapses (VD3–VD11) labeled with ELKS-1::tdTomato, density (puncta/10 μm) in wild-type (3.33 ± 1.1), *unc-55* (0.99 ± 0.9), *unc-55; unc-8* (0.23 ± 0.2), *unc-55* control RNAi (0.99 ± 1.2), and *unc-55; irx-1* RNAi (2.12 ± 1.4) L4 stage larvae. Data are mean ± SD. Kruskal–Wallis test with multiple comparison because *unc-55*, *unc-55;unc8*, and *unc-55;* control RNAi samples are not normally distributed; *N* > 16 animals; *p < 0.05, **p < 0.01, ****p < 0.0001. NS = not significant. ***H***, Representative images of VD10 neuron in control (left) versus VD10 with forced UNC-8 expression [UNC-8(OE)] (right). Puncta are *unc-8* smFISH probe (magenta) and nuclei are labeled with DAPI (blue). Dashed outlines denote VD10 cell soma marked with P*unc-25*::GFP. Scale bar: 5 µm. ***I***, Violin plots for *unc-8* smFISH puncta in control (white; *n* = 42 cells) versus UNC-8(OE) (green; *n* = 45 cells) in L3 stage VD motor neurons confirm elevated *unc-8* transcripts in UNC-8(OE) VD neurons. Dashed line denotes median. Mann–Whitney test was used to determine significance because the control sample was not normally distributed, ****p < 0.0001. ***J***, top, Representative image of ELKS-1::TdTomato (magenta) in the ventral nerve cord of mosaic animals with forced UNC-8 expression [UNC-8(OE)] in a subset of VD neurons (dashed outline, green, VD10) versus neighboring control VD neurons (dashed outline, VD11). Arrowheads point to ELKS-1::TdTomato puncta in control and UNC-8(OE) VD neurons. Dashed circles demark VD cell soma. Below, Schematic of ELKS-1 puncta (magenta) in control (gray) and UNC-8(OE) cells (green). Scale bar: 5 μm. Images from L4 animals, anterior to left. ***K***, Forced expression of UNC-8 in VD neurons [UNC-8(OE)] (1.00 ± 0.6) does not reduce ELKS-1::tdTomato levels in VD presynaptic terminals compared with neighboring control VD neurons that do not express UNC-8 (1.38 ± 0.8). Data are mean ± SD; *N* = 15. NS = not significant. Paired *t* test, *p* = 0.0923. ***L***, Working model: IRX-1 drives expression of UNC-8 and also activates an additional pathway (?) that removes UNC-13 and ELKS-1 from remodeling GABAergic synapses. ***M***, Schematic of components that are selectively removed from remodeling GABAergic presynaptic domains by UNC-8 (green) or by IRX-1 (green + magenta). Plasma-membrane SNAREs (gray) are shown because these are required for functional synapses (Fig. 5*G*).

Based on previous work demonstrating that ELKS-1 recruits the mammalian protein bMunc-13–2 to active zones and that *Drosophila* ELKS homolog, Bruchpilot, recruits UNC-13L/Unc13A (Böhme et al., 2016; Kawabe et al., 2017), we next asked whether ELKS-1 is also removed by IRX-1/Iroquois. We determined that expression of ELKS-1::tdTomato (Cherra and Jin, 2016) in wild-type adult GABA neurons results in bright fluorescent puncta characteristic of DD synapses in the dorsal nerve cord and VD synapses in the ventral nerve cord (Fig. 8*A*). Ventral ELKS-1::tdTomato-labeled puncta are largely absent in *unc-55* mutants indicating that ELKS-1 is dismantled from the presynaptic domains of remodeling GABAergic motor neurons (Fig. 8*C*,*G*). Ventral ELKS-1 puncta are also missing in *unc-55; unc-8* mutants (Fig. 8*D*,*G*). Thus, UNC-8 function is not required to remove ELKS-1 from the presynaptic domain. Surprisingly, the ventral ELKS-1 signal is reduced in *unc-55; unc-8* animals relative to *unc-55.* However, the significance of this finding is unclear because we do not observe a similar negative effect of the *unc-8* mutation on ELKS-1 levels in remodeled DD neurons (Fig. 10*D*).

In contrast to results obtained from *unc-55; unc-8* mutants (Fig. 8*D*,*G*), a substantial number of ELKS-1 puncta are retained in the ventral nerve cord of *unc-55* animals treated with *irx-1* RNAi (Fig. 8*F*,*G*). This finding suggests that IRX-1/Iroquois promotes the removal of ELKS-1 in remodeling GABAergic neurons but that UNC-8 is not required. For a direct test of this model, we used a transgenic strategy to overexpress UNC-8 in VD neurons (Fig. 8*H*,*I*). Although UNC-8(OE) in VD neurons drives the removal of ventral SNB-1::GFP (Fig. 7*H*,*I*), we observed that UNC-8(OE) is not sufficient to eliminate ELKS-1 (Fig. 8*J*,*K*). Thus, our results support the idea that Iroquois/IRX-1 removes ELKS-1 from ventral synapses of remodeling GABAergic motor neurons in a genetic pathway that is independent of UNC-8 (Fig. 8*L*,*M*).

To summarize, our results show that IRX-1 drives the removal of multiple components of the presynaptic apparatus in remodeling GABAergic neurons including Synaptobrevin/SNB-1, RAB-3, liprin-α/SYD-2, Endophilin/UNC-57, UNC-13L, and ELKS-1 (Figs. 3, 4, 7, 8). In contrast, DEG/ENaC/UNC-8 promotes the disassembly of Synaptobrevin/SNB-1, RAB-3, liprin-α/SYD-2, and Endophilin/UNC-57 (Fig. 3) but is not required for the removal of Munc-13/UNC-13L and ELKS-1 (Figs. 7, 8).

### Iroquois/IRX-1 and DEG/ENaC/UNC-8 drive the removal of RAB-3 from remodeling DD neuron GABAergic synapses

**Figure 9.**
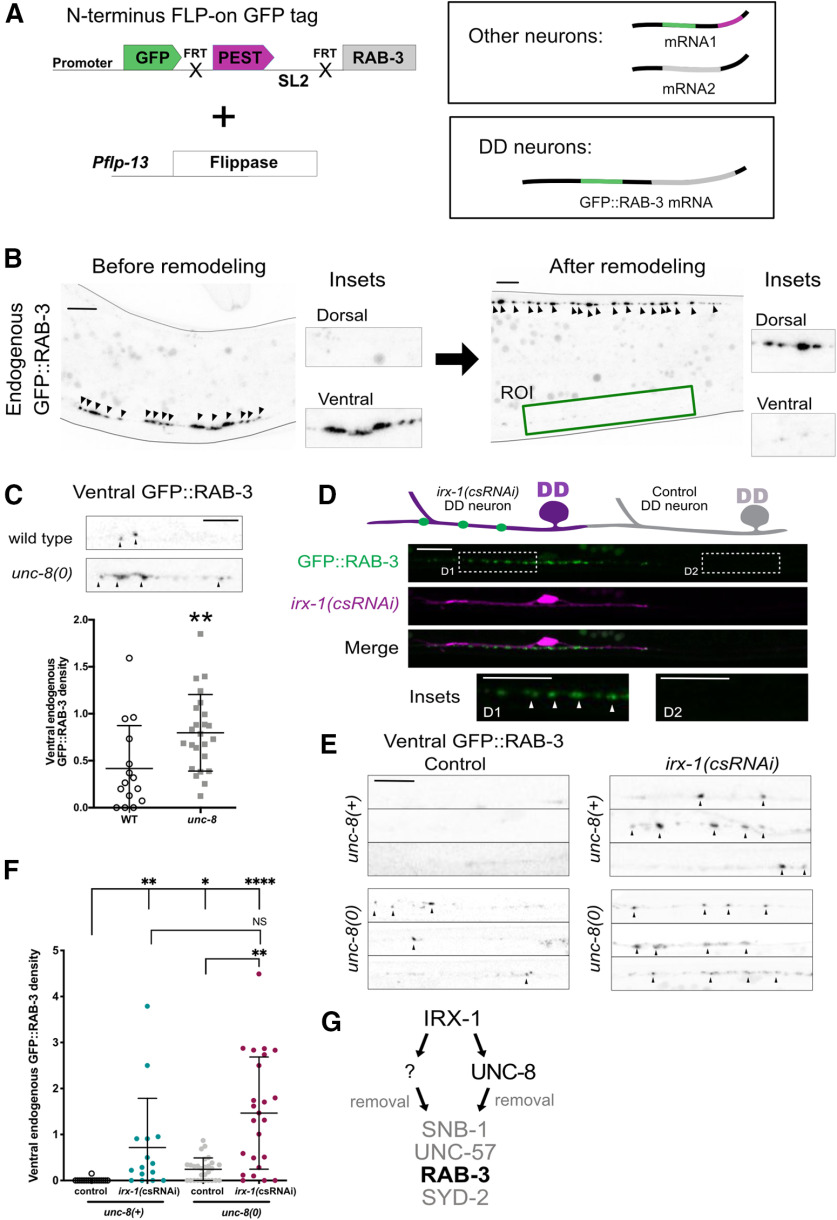
IRX-1 activates parallel pathways that remove RAB-3 from the ventral terminals of remodeling DD neurons. ***A***, Endogenous labeling of RAB-3 with GFP in DD neurons. *Pflp-13* drives flippase expression to attach GFP to the N terminus of the endogenous RAB-3 protein in DD neurons (Schwartz and Jorgensen, 2016). ***B***, Fluorescent images of endogenous GFP::RAB-3 in DD neurons in the ventral nerve cord before remodeling (left) and in the dorsal nerve cord after remodeling (right). Green box denotes region of interest (ROI) in L4 stage larva for counting DD-specific ventral GFP::RAB-3 puncta. Arrowheads point to GFP::RAB-3 puncta. Scale bar: 5 μm. ***C***, top, Representative images of ventral nerve cords of wild-type and *unc-8* mutant L4 stage larvae. Arrowheads denote ventral GFP::RAB-3 puncta. Scale bar: 5 μm. Bottom, *unc-8* mutants (0.80 ± 0.4, *n* = 24) retain more GFP::RAB-3 puncta than wild type (0.41 ± 0.5, *n* = 15). Data are mean ± SD. Unpaired *t* test; **p = 0.0051. Ventral puncta in DD2 and DD3 were scored for this analysis. ***D***, top, Schematic of ventral GFP::RAB-3 puncta (green) retained at the L4 larval stage in *irx-1(csRNAi)* DD neurons (magenta) but not in neighboring DD neurons (control) that do not express *irx-1(csRNAi)* (gray). Bottom, Representative image of *irx-1(csRNAi)*-expressing DD2 neuron (magenta) that retains ventral GFP::RAB-3 (green) and DD3 (control) in which GFP::RAB-3 puncta are removed from the ventral nerve cord. D1 inset shows GFP::RAB-3 puncta (arrowheads) that remain in the ventral nerve cord of DD neurons with *irx-1(csRNAi)* knock-down. Neighboring D2 inset shows control DD neuron that remodels and eliminates GFP::RAB-3 puncta. Scale bar: 10 μm. ***E***, Representative images of ventral regions of control and *irx-1(csRNAi)* DD neurons in *unc-8(*+*)* (top) and *unc-8(0)* mutant (bottom) L4 stage larvae. Arrowheads denote GFP::RAB-3 puncta. Scale bar: 5 μm. Note that *irx-1(csRNAi)* enhances retention of GFP-RAB-3 puncta in *unc-8* mutants. ***F***, *irx-1(csRNAi)* enhances retention of GFP::RAB-3 puncta in *unc-8* mutants. Density (puncta/10 μm) of GFP::RAB-3 puncta in the ventral nerve cords of control (0.01 ± 0.03, *n* = 17) and *irx-1(csRNAi)* (0.72 ± 1.1, *n* = 15) DD neurons in control (gray; 0.24 ± 0.3, *n* = 28) and *irx-1(csRNAi)* (1.47 ± 1.2, *n* = 24) in *unc-8(*+*)* (left) versus *unc-8(0)* mutant (right) backgrounds. Data are mean ± SD. Non-parametric Kruskal–Wallis test because control datasets are not normally distributed; *p < 0.05, **p < 0.01, ****p < 0.0001; and NS = not significant. ***G***, Working model: in DD neurons, IRX-1 activates UNC-8 and a parallel pathway (?) to remove RAB-3 (bold) from the ventral terminals of remodeling DD neurons. Additional presynaptic components (SNB-1, UNC-57, SYD-2) are removed from ventral GABAergic boutons in *unc-55* mutants in which both DD and VD neurons remodel.

We have exploited the ectopic remodeling phenotype of *unc-55* mutant VD neurons to show that both IRX-1 and UNC-8 act to dismantle the presynaptic apparatus. In addition, our findings suggest that IRX-1 functions as a transcription factor to orchestrate the overall mechanism by activating expression of UNC-8 as well as another downstream pathway that functions in parallel to drive synapse removal (Fig. 8*L*). To determine whether a similar mechanism also drives synapse elimination in the native DD remodeling program, we used an endogenous GFP::RAB-3 marker that is specifically expressed in DD neurons (Fig. 9*A*). As expected, in the wild-type, endogenous RAB-3::GFP remodels from ventral to dorsal locations during early larval development; few ventral GFP::RAB-3 puncta are detectable by the L4 larval stage (Fig. 9*B*). In contrast, *unc-8* mutants at the L4 stage show significant retention of GFP::RAB-3 signal (Fig. 9*C*), likely resulting from failed synapse elimination (Miller-Fleming et al., 2016). Similarly, csRNAi of *irx-1* in DD neurons prevents the elimination of GFP::RAB-3 (Fig. 9*D–F*), indicating that IRX-1 is also necessary for the efficient removal of presynaptic GFP::RAB-3 in DD neurons (Petersen et al., 2011). Notably, significantly more GFP::RAB-3 puncta are retained in *irx-1(csRNAi)-*treated DD neurons than in *unc-8* mutants (Fig. 9*F*), a result consistent with the idea that IRX-1 drives expression of both *unc-8* and an additional pathway for synapse elimination. This model of parallel-acting pathways predicts that *irx-1(csRNAi)* should enhance the synaptic removal defect of *unc-8* mutants, which we also observe (Fig. 9*E*,*F*). Finally, genetic ablation of *unc-8* does not enhance retention of residual GFP::RAB-3 by *irx-1(csRNAi)* (Fig. 9*F*), a result consistent with the idea the IRX-1 is required for *unc-8* expression.

To summarize, our results have confirmed that (1) IRX-1 and UNC-8 promote the elimination of RAB-3 from ventral terminals of DD neurons (Fig. 9); (2) IRX-1 functions upstream of UNC-8 to remove RAB-3 (Fig. 2); and (3) IRX-1 controls an additional pathway that disassembles RAB-3 independently of UNC-8 (Fig. 9*E*,*F*). Because these findings were also observed for ectopically remodeling VD neurons (Figs. 3, 4), we propose that the additional presynaptic components, Synaptobrevin/SNB-1, Endophilin/UNC-57, and liprin-α/SYD-2, are similarly regulated during DD synapse removal (Fig. 9*G*).

### Iroquois/IRX-1, but not DEG/ENaC/UNC-8, removes ELKS-1 from ventral synapses in DD neurons

**Figure 10.**
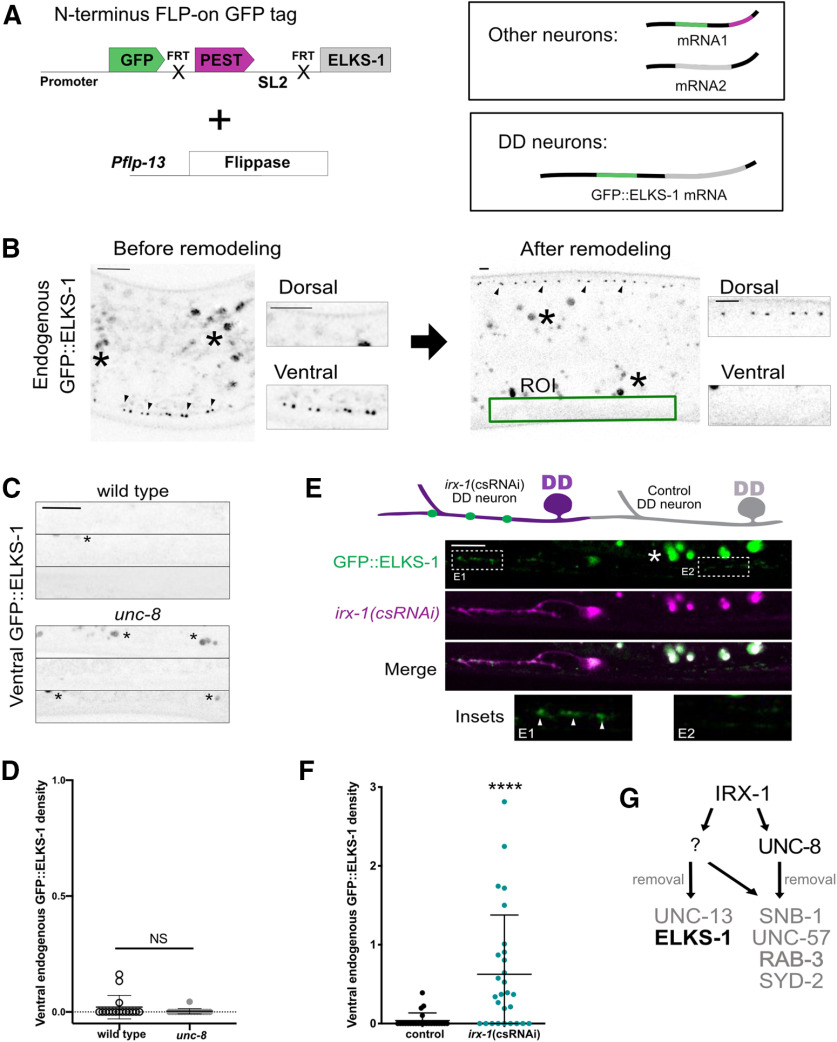
IRX-1, but not UNC-8, drives removal of endogenous ELKS-1 from ventral terminals of remodeling DD neurons. ***A***, Endogenous labeling of ELKS-1 with GFP in DD neurons. *pflp-13* drives flippase in DD neurons to fuse GFP to the N terminus of the endogenous ELKS-1 protein (Schwartz and Jorgensen, 2016). ***B***, Fluorescent images of endogenous GFP::ELKS-1 in DD neurons in the ventral nerve cord before remodeling (left) in L1 larvae versus the dorsal nerve cord after remodeling (right) at the L4 stage. Green box defines region of interest (ROI) for counting DD-specific ventral GFP::ELKS-1 puncta (arrowheads). Asterisks denote autofluorescence. Scale bar: 5 μm. ***C***, ***D***, Representative images of ventral nerve cords of wild-type (0.020 ± 0.05, *n* = 16) and *unc-8* mutant (0.003 ± 0.01, *n* = 15) L4 stage larvae which eliminate GFP::ELKS-1; density = puncta/10 μm. Asterisks denote autofluorescence. Data are mean ± SD. Mann–Whitney test, NS is not significant, *p* = 0.1913. Scale bar: 5 μm. ***E***, top, Schematic of ventral GFP::ELKS-1 puncta (green) retained at the L4 larval stage in *irx-1(csRNAi)* treated DD neurons (magenta) but not in neighboring DD neurons (control) that do not express *irx-1(csRNAi)* (gray). Bottom, Representative image of DD1(*irx-1(csRNAi)* that retains ventral GFP::ELKS-1 puncta (green) and DD2 (control) that eliminates GFP::ELKS-1 puncta from the ventral nerve cord (green). ***E1*** inset, Residual ventral GFP::ELKS-1 puncta that remain in the DD1 neuron with *irx-1(csRNAi)* knock-down. Neighboring ***E2*** inset, Control DD2 neuron that remodels and eliminates ventral GFP::ELKS-1 puncta. Scale bar: 5 μm. ***F***, GFP::ELKS-1 puncta (0.63 ± 0.8, *n* = 28) are elevated in *irx-1(csRNAi)* DD neurons at the L4 larval stage compared with control DD neurons that undergo remodeling (0.04 ± 0.1, *n* = 24). density = puncta/10 μm. Data are mean ± SD. Mann–Whitney test, ****p < 0.0001. ***G***, Working model: IRX-1, but not UNC-8, removes ELKS-1 from the ventral terminals of remodeling DD neurons and UNC-13 in *unc-55* mutants in which both DD and VD neurons remodel (Fig. 8).

Our results obtained from ectopic remodeling of VD neurons in *unc-55* mutants showed that IRX-1, but not UNC-8, promotes removal of a specific subset of active zone proteins, ELKS-1 and UNC-13 (Figs. 7, 8). To determine whether ELKS-1 is similarly regulated in remodeling DD neurons, we used an endogenous GFP::ELKS-1 marker that is selectively expressed in DD neurons (Fig. 10*A*). In the wild-type, GFP::ELKS-1 is initially deposited at ventral DD synapses and then relocated to dorsal DD synapses as predicted for the DD remodeling program (Fig. 10*B*). GFP::ELKS-1 is also removed from ventral DD synapses of *unc-8* mutants (Fig. 10*C*,*D*), suggesting that UNC-8 is not required for ELKS-1 elimination. In contrast, we observed that *irx-1* knock-down by *irx-1(csRNAi)* antagonizes the elimination of GFP::ELKS-1 from ventral synapses of DD neurons (Fig. 10*E*,*F*). Thus, our results demonstrate that IRX-1 but not UNC-8 drives ELKS-1 removal in DD neurons. Because Munc13/UNC-13 is also selectively removed by IRX-1 in ectopically remodeling VD neurons (Fig. 7), we propose that Munc13/UNC-13 is similarly regulated in remodeling DD neurons (Fig. 10*G*). Our findings are notable because they show that distinct genetic pathways can dismantle the presynaptic apparatus in remodeling GABAergic neurons by targeting specific active zone components.

## Discussion

### Presynaptic disassembly in remodeling in GABAergic neurons

**Figure 11.**
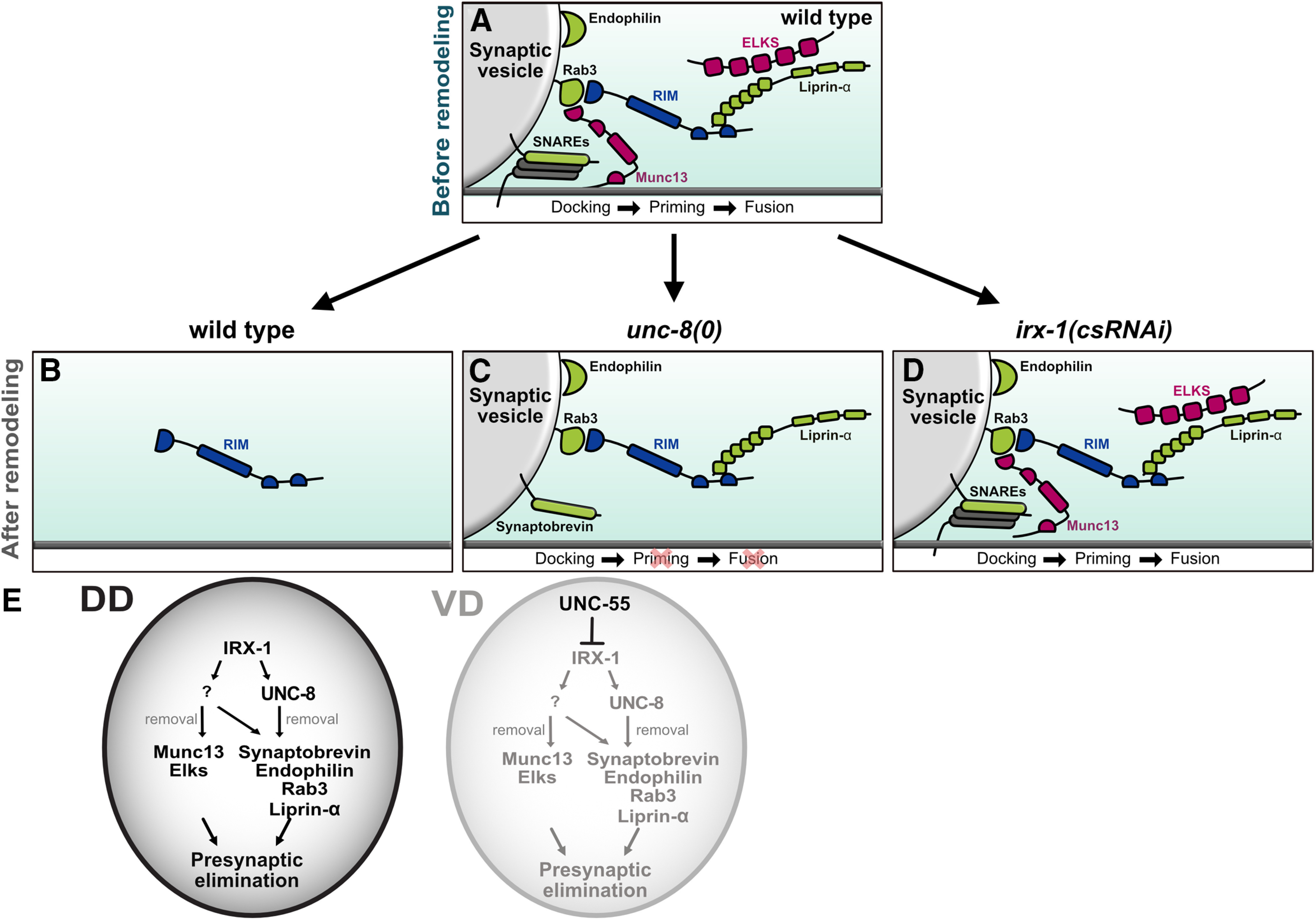
Parallel-acting pathways dismantle the presynaptic apparatus in remodeling GABAergic neurons. ***A***, top, Before remodeling, presynaptic proteins SNAREs, Rab3, RIM, endophilin, liprin-α, Munc13, and ELKS mediate SV fusion and neurotransmitter release on the ventral side of GABAergic neurons. ***B***, After remodeling, presynaptic markers except RIM are removed from the ventral nerve cord. ***C***, Synaptobrevin, Rab3, RIM, endophilin, and liprin-α are retained at ventral presynaptic regions of GABAergic neurons in *unc-8* mutants. Note that *unc-8* activity is not required for removal of ELKS and Munc13. ***D***, With RNAi knock-down of the Iroquois/IRX-1 transcription factor, Synaptobrevin, Rab3, RIM, endophilin, liprin-α, Munc13, and ELKS persist in the ventral nerve cord to mediate GABA release from functional terminals. Plasma-membrane SNAREs are depicted because these synapses are functional. ***E***, Transcriptional regulation of parallel-acting pathways that drive presynaptic disassembly. In DD neurons (left), the transcription factor IRX-1/Iroquois activates expression of at least two downstream targets. (1) The DEG/ENaC channel subunit, UNC-8, which promotes removal of Synaptobrevin, endophilin, Rab3 and liprin-α but not the active zone proteins Munc13 or ELKS. (2) A second pathway (?), that promotes removal of Synaptobrevin, endophilin, Rab3 and liprin-α as well as Munc13 and ELKS. In VD neurons (right), the COUP-TF transcription factor UNC-55 blocks expression of IRX-1 thereby preventing the elimination of ventral presynaptic terminals.

Synaptic plasticity is a key dynamic feature of the nervous system as neurons actively assemble new synapses while also dismantling others (Linkenhoker et al., 2005; De Paola et al., 2006; Stettler et al., 2006; Hong et al., 2014; Mcbride and Debello, 2015). In contrast to synaptic assembly about which much is known, the molecular mechanisms that drive synaptic elimination are relatively unexplored (Südhof, 2017, 2018). In this study, we investigated a developmentally-regulated mechanism of presynaptic disassembly in *C. elegans* (White et al., 1978; Hallam and Jin, 1998; Cuentas-Condori and Miller, 2020). Our findings revealed parallel-acting pathways that selectively remove different components of the presynaptic active zone in remodeling GABAergic synapses (Fig. 11). We have shown that the conserved transcription factor Iroquois/IRX-1 drives expression of the DEG/ENaC channel subunit UNC-8 (Fig. 2) to remove the presynaptic proteins Synaptobrevin/SNB-, liprin-α/SYD-2, Endophilin/UNC-57, and RAB-3 (Figs. 3, 9; Miller-Fleming et al., 2016). IRX-1 regulates a parallel-acting mechanism that also removes these presynaptic components (Fig. 4; Petersen et al., 2011). In addition, Iroquois/IRX-1 promotes the selective disassembly of ELKS-1 and Munc13/UNC-13 in a separate mechanism that does not require UNC-8 activity (Figs. 7, 8, 10). Together, our findings show that synaptic disassembly can be transcriptionally-regulated and involve molecularly distinct mechanisms that differentially eliminate selected subsets of presynaptic proteins.

### Activity-dependent active zone remodeling

The active zone region of the presynaptic terminal mediates SV fusion for neurotransmitter release (Südhof, 2012). This active zone function is defined by a core group of components including VGCCs, ELKS, Munc13/UNC-13, liprin-α/SYD-2, SYD-1, RIM/UNC-10, and RBP (Südhof, 2012). Notably, the composition and size of the SV release machinery can be modulated by synaptic activity. For example, additional copies of specific active zone proteins (i.e., ELKS, RBP, VGCCs, and Munc13) are incorporated into the presynaptic zones of *Drosophila* neuromuscular junctions (NMJs) in a homeostatic mechanism that elevates neurotransmitter release to compensate for reduced postsynaptic sensitivity (Böhme et al., 2019; Gratz et al., 2019). Elevated activity in *Drosophila* photoreceptors can also have the opposite effect of selectively removing a subset of these presynaptic proteins (liprin-α, RIM, and RBP) while leaving others intact (VGCCs and SYD-1) to adapt the synapse to different sensory inputs (Sugie et al., 2015).

Our findings point to a related effect in remodeling GABAergic neurons in *C. elegans* in which neuronal activity promotes the elimination of selected presynaptic components. In previous work, we determined that the DEG/ENaC channel UNC-8 functions in an activity-dependent pathway that dismantles the presynaptic active zone. Genetic results, for example, show that UNC-8 acts in a common pathway with the VGCC, UNC-2 (Miller-Fleming et al., 2016). Thus, we propose here that UNC-8 drives the removal of a core group of presynaptic proteins including; Synaptobrevin/SNB-1, liprin-α/SYD-2, Endophilin/UNC-57, and RAB-3, which depends on GABA neuron activity and cytoplasmic calcium. In contrast, synaptic elimination of Munc13 and ELKS does not require UNC-8 and is selectively regulated in a separate pathway driven by the transcription factor Iroquois/IRX-1. Additionally, our studies show that IRX-1 drives disassembly of the same core group of presynaptic proteins (Synaptobrevin/SNB-1, liprin-α/SYD-2, Endophilin/UNC-57, RAB-3; Fig. 11). Although UNC-8 is a key downstream effector for this mechanism, our genetic evidence also indicates that Iroquois/IRX-1 must regulate at least one additional downstream gene to remove this core group of components from the presynaptic region (Figs. 4, 9). Future studies are needed to define the additional downstream IRX-1 effectors that drive presynaptic disassembly. These IRX-1 targets could emerge from previously defined datasets of genes regulated by the UNC-55/COUP-TF transcription factor (Petersen et al., 2011; Yu et al., 2017) since UNC-55 controls IRX-1 expression in VD GABAergic neurons (Fig. 11; Petersen et al., 2011; He et al., 2015).

### Presynaptic domains are remodeled within intact axons

We have described an example of activity-dependent circuit refinement in *C. elegans* in which presynaptic termini are eliminated in a mechanism that does not perturb axonal morphology (White et al., 1978). Presynaptic domains are also selectively dismantled from intact axons in activity-dependent mechanisms that sculpt the developing mammalian visual circuit. Initially, retinal ganglion cells (RGCs) extend exuberant axonal projections to the lateral geniculate nucleus. Later, RGC inputs to each geniculate neuron are reduced. During this period, axonal pruning for at least one class of RGCs (BD-RGCs) is preceded by the internal reorganization of presynaptic boutons which are eliminated in distal axonal regions and simultaneously assembled in proximal locations (Hong et al., 2014). Axons denuded of presynaptic domains are then retracted in a later phase of refinement (Hong and Chen, 2011). Inputs to RGCs from rod bipolar cells (BCs) in the retina are also eliminated from stable axonal-dendritic contacts during development (Morgan et al., 2011). Thus, the reorganization of presynaptic domains within intact RGC and BC axons is similar to the remodeling mechanism in *C. elegans* GABAergic neurons in which the presynaptic apparatus is dismantled without visible alterations in axonal morphology (White et al., 1978; Hallam and Jin, 1998). Presynaptic boutons are also actively assembled as well as removed within intact axonal processes in the adult brain (De Paola et al., 2006; Stettler et al., 2006). Notably, key components involved in presynaptic remodeling in *C. elegans* GABAergic neurons are highly conserved (Fig. 11). Together, these findings suggest that the molecular pathways that control presynaptic remodeling in *C. elegans* may also regulate circuit refinement and plasticity in mammals.

### Remodeling and “silent” synapses

Our work has revealed a synaptic remodeling mechanism that disables neurotransmitter release while leaving SVs and the presynaptic density intact. We showed, for example, that proteins with essential roles in SV priming, Munc13/UNC-13 and ELKS, can be selectively removed from the presynaptic apparatus in a genetic background that preserves the ultrastructural integrity of the active zone (Fig. 4*A*); thus, effectively “silencing” an otherwise normal appearing synapse (Fig. 5*C*). A potentially related phenomenon of synaptic silencing has been reported in the auditory circuit of the barn owl. Juvenile owls fitted with optical prisms learn to associate auditory cues with a new imposed visual location (Knudsen and Knudsen, 1989). Adaptation in this case involves innervation of a new midbrain region in the auditory localization circuit. Synaptic boutons are also maintained, however, in the nearby anatomic domain in which object association normally occurs (Mcbride et al., 2008) which could account for the restoration of normal responses to auditory cues in adult owls after the training prisms are removed (Mcbride and Debello, 2015). The retention of these inactive synaptic structures could correspond to more broadly observed “learning traces” that facilitate the ready reacquisition of quiescent behavioral responses (Knudsen, 2002). We thus suggest that the elucidation of mechanisms that disable synapses by removing specific functional components could reveal the molecular underpinning of presynaptic silencing mechanisms with key roles in learning and memory circuits.
